# Meta-analysis of transcriptomic data reveals clusters of consistently deregulated gene and disease ontologies in Down syndrome

**DOI:** 10.1371/journal.pcbi.1009317

**Published:** 2021-09-27

**Authors:** Ilario De Toma, Cesar Sierra, Mara Dierssen

**Affiliations:** 1 Centre for Genomic Regulation, The Barcelona Institute of Science and Technology, Barcelona, Spain; 2 Universitat Pompeu Fabra, Barcelona, Spain; 3 Centro de Investigación Biomédica en Red de Enfermedades Raras, Barcelona, Spain; University College London, UNITED KINGDOM

## Abstract

Trisomy of human chromosome 21 (HSA21) causes Down syndrome (DS). The trisomy does not simply result in the upregulation of HSA21--encoded genes but also leads to a genome-wide transcriptomic deregulation, which affect differently each tissue and cell type as a result of epigenetic mechanisms and protein-protein interactions.

We performed a meta-analysis integrating the differential expression (DE) analyses of all publicly available transcriptomic datasets, both in human and mouse, comparing trisomic and euploid transcriptomes from different sources. We integrated all these data in a “DS network”.

We found that genome wide deregulation as a consequence of trisomy 21 is not arbitrary, but involves deregulation of specific molecular cascades in which both HSA21 genes and HSA21 interactors are more consistently deregulated compared to other genes. In fact, gene deregulation happens in “clusters”, so that groups from 2 to 13 genes are found consistently deregulated. Most of these events of “co-deregulation” involve genes belonging to the same GO category, and genes associated with the same disease class. The most consistent changes are enriched in interferon related categories and neutrophil activation, reinforcing the concept that DS is an inflammatory disease. Our results also suggest that the impact of the trisomy might diverge in each tissue due to the different gene set deregulation, even though the triplicated genes are the same.

Our original method to integrate transcriptomic data confirmed not only the importance of known genes, such as SOD1, but also detected new ones that could be extremely useful for generating or confirming hypotheses and supporting new putative therapeutic candidates. We created “metaDEA” an R package that uses our method to integrate every kind of transcriptomic data and therefore could be used with other complex disorders, such as cancer. We also created a user-friendly web application to query Ensembl gene IDs and retrieve all the information of their differential expression across the datasets.

## Author summary

We analyzed all publicly available transcriptomic datasets on Down syndrome, the most common genetic intellectual disability, caused by trisomy of chromosome 21. Even though the triplicated genes are the same, the resulting transcriptional profile differs according to different tissues and states. Therefore, our goal was to understand which genes are most consistently deregulated both within and across different tissue macro-categories. We found that the effects of gene deregulation in Down syndrome originate from the upregulation of the triplicated genes but extend to others that are regulated or interact with the ones encoded on chromosome 21. This creates clusters of genes possibly involved in common biological or pathological processes, that are differentially expressed at the same time in different tissues or cellular states (co-DE expressed genes). The most consistent changes were specifically enriched in interferon related categories and neutrophil activation, reinforcing the concept that DS is an inflammatory disease. The genes that we detected can be very important for projects targeting key processes in Down syndrome for therapeutic purposes, as we show with the previously unreported upregulation of *ETNPPL* upon learning. Moreover, by applying our approach to different transcriptomic datasets, our method could be used for other complex disorders, such as cancer. This has been made easy thanks to development of “metaDEA”, an intuitive R package available on github.

## Introduction

Down syndrome (DS) is the most common genetic form of intellectual disability caused by the complete or partial trisomy of human chromosome 21 (HSA21)[1]. This results in a complex syndrome where intellectual disability, one of its main hallmarks, goes along with a series of phenotypes that affect the whole organism. Having three copies instead of two for each HSA21 gene would lead theoretically to an increased transcription by a factor of 1.5 [2]. This dosage imbalance hypothesis is supported by mouse models overexpressing single dosage sensitive genes such as *DYRK1A* [3], *SOD1* [4], or *HMGN1* [5] that recapitulate part of the DS phenotype. However, transcriptomic studies revealed that triplication of HSA21 does not simply translate into overexpression of HSA21 genes but leads to a genome wide transcriptional deregulation [6]. In fact, we now know that gene expression is not simply proportional to copy number. Genes are transcribed with different efficiency due to the levels of transcription factors and to the epigenetic state that influence chromatin accessibility.

Despite the large number of studies on DS, genome-wide deregulation in the different tissues is still not completely understood. Are there some genes that tend to be deregulated in all tissue types? Or has every tissue a specific pattern of deregulation? In an effort to shed light into the transcriptomic alterations in DS, we performed a meta-analysis of all available transcriptomic data.

**Fig 1:**
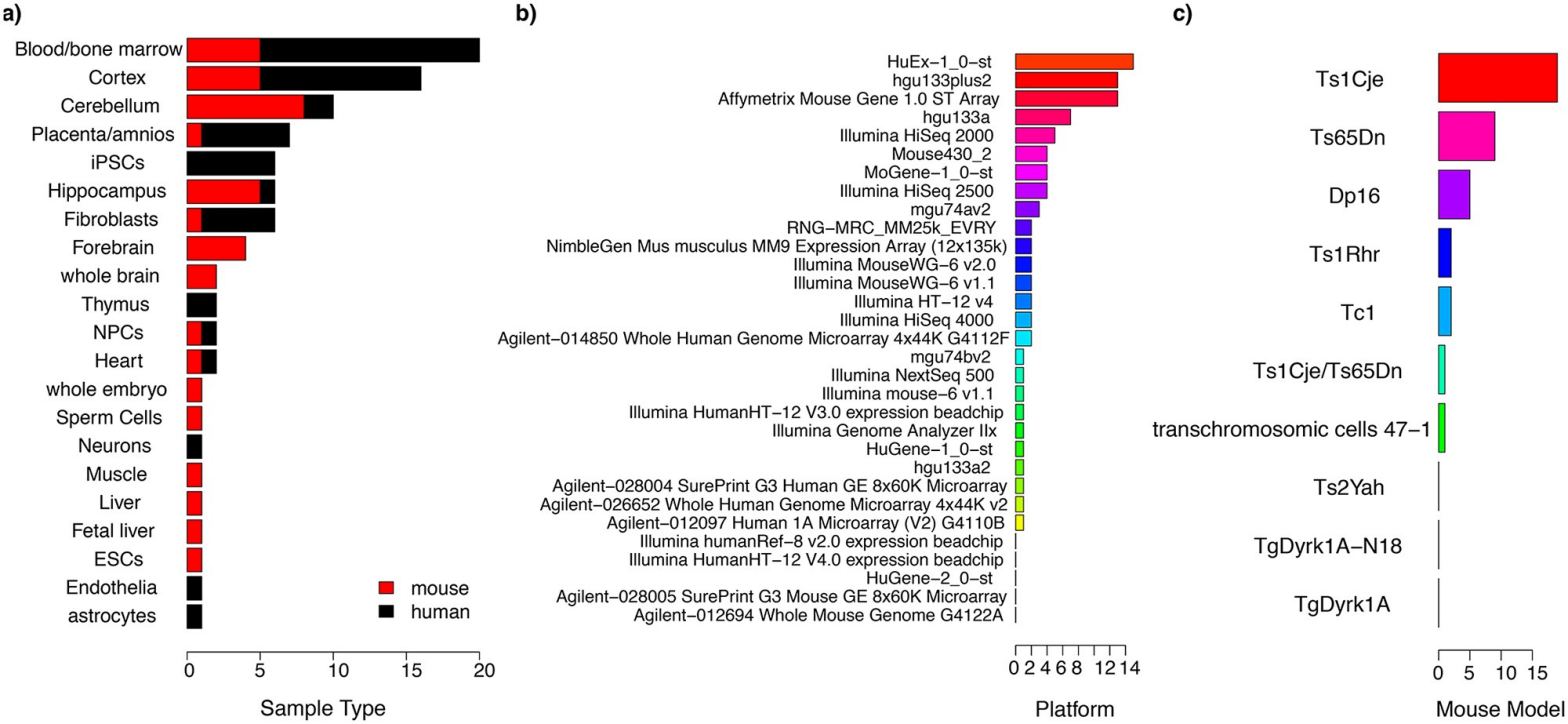
Transcriptomic data used for the meta-analysis. The datasets used derive from both individuals with DS and DS mouse models, and span different tissues, sex, ages and treatment effects. **a**) Barplot showing the number of transcriptomic datasets for each tissue in *Mus Musculus* (red) and *Homo sapiens* (black). **b)** Number of transcriptomic datasets using each sequencing platform. **c)** Number of transcriptomic datasets for each DS mouse model.

A meta-analysis from heterogeneous DS data was previously published [7] but it only included microarray and proteomic experiments published before 2010. We therefore performed the most comprehensive meta-analysis on transcriptomic DS data to date, including all publicly available microarray and RNAseq datasets in mouse and human (Fig 1 and S1 Table). Using these datasets, we created a DS gene deregulation network, by selecting the most consistently differentially expressed (DE) genes across studies. Our analysis revealed that genes mapping on HSA21 and their interactors tended to be deregulated across different tissues, while non-HSA21 genes were less consistently DE. Interestingly, genes co-DE often belonged to the same molecular process or were associated with the same disease ontologies. Our meta-analysis also shed light on previously unreported DS genes, such as *Etnppl*, that we found upregulated upon learning in wild type but not in trisomic mice. We also created a user-friendly web-application to query specific ensembl gene IDs and get their DE data across the databases.

## Results

### Down syndrome transcriptomic meta-analysis

We performed a meta-analysis using all available transcriptomic data (67 studies, 56 from published articles by 24/01/2020) comparing trisomic versus euploid samples of human and mouse (Fig 1 and S1 Table). The aim was to identify genes that are differentially expressed (DE) across different experiments. The datasets derive from different tissues and organs (e.g. for the brain there were datasets of cerebral cortex, hippocampus, etc.; for blood there were dataset of lymphocytes, T-cells, etc.). Therefore, we grouped all the comparisons in tissue macro-categories: "Blood/bone marrow", "Brain”, “Endothelial", "Fibroblasts", "Heart", "Liver", "Muscle", "Placenta/amnios", and "Undifferentiated" (Fig 1A). The datasets include microarray and RNA-seq experiments performed in different platforms (Fig 1B). The datasets from *Mus Musculus* are from different mouse models, the most frequent being the Ts1Cje mice, followed by the Ts65Dn (Fig 1C).

**Fig 2:**
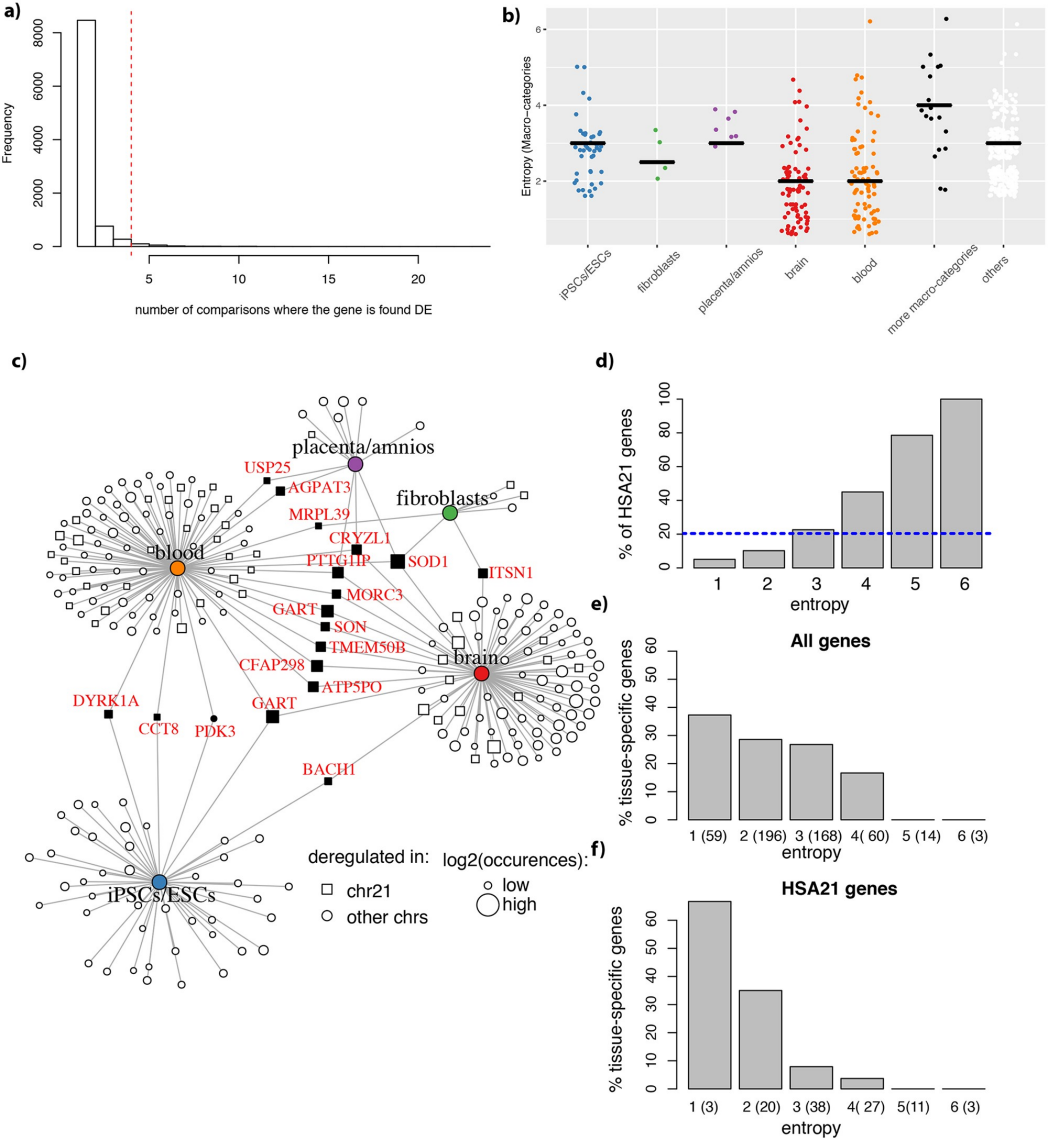
Tissue macro-categories show both HSA21 and non-HSA21 genes that are consistently DE. **a)** Histogram of DE genes’ occurrences. Genes in the right 5% are in the right of the red dot line, corresponding to genes found DE in at least 4 comparisons **b)** Entropy distribution across consistently DE genes. Each dot is a gene, the horizontal bar indicates the median entropy for each group. All genes consistently DE in iPSCs/ESCs were also found DE in at least another macro-category up to 4 other macro categories. Gene consistently DE in fibroblasts were also found DE in one or two macro-categories. Similarly, genes consistently DE in placenta/amnios were found DE also in 2 or 3 other macro categories. For brain and blood tissue we did detect a fraction specifically DE only in one of the two macro-categories, however, also in this case we detected genes DE in more macro-categories (1 to 4 for brain; 1 to 5 for blood). In black are genes that are consistently DE at the tissue level in more than one macro-category. In white, genes that are not consistently DE in any specific macro categories but are consistently DE across tissue. **c)** Bipartite graph showing the tissue macro-categories where we could find consistently DE genes at the tissue level. For each tissue macro-category, we plotted the frequency of the number of occurrences. The red dotted line indicates the minimal number of occurrences chosen for a gene to be DE in a given tissue macro-category. Genes mapping on HSA21 are represented by squares, otherwise by circles. Node size is proportional to the log_2_ of the number of occurrences across all comparisons. Of note, most consistently DE genes are HSA21. **d)** Barplot for the percentage (%) of HSA21 genes for each entropy level. The dotted line indicates the percentage (%) of HSA21 genes in the top-500 DE genes. **e)** Barplot showing the percentage (%) of tissue specific genes for each entropy level, considering all the top 500 genes f) As in e). but, only considering the top 500 genes mapping on HSA21.

The studies encompassed a total of 125 comparisons. In fact, each study could contain comparisons of disomic versus trisomic samples of different conditions like tissues, sex, and age. For each comparison, we performed a differential expression (DE) analysis blocking for all factors but trisomy (see Methods). When filtering for adjusted p-value (<0.05) and fold change (1.5 fold) we remained with 51 studies (45 of them published) for a total of 92 comparisons (since the other comparisons had no significant genes passing those thresholds). We detected 51902 genes across all comparisons and found 9727 genes DE in at least one trisomic versus euploid comparison. Given the high number of comparisons, we expected many genes to be false positives, or specific to one particular experiment. Thus, to identify bona fide genes, we selected those most consistently DE across all comparisons. In this way we identified 500 genes that were DE in at least 4 comparisons (5% of the distribution inferred for the histogram in Fig 2a). The analyses performed are summarized in S1 Fig.

We calculated the entropy of a given gene, defined as the number of tissue macro-categories in which it was DE (S2 Fig). For example, a gene appearing in four comparisons from blood and one from the brain has an entropy of 2. In this way, we found that most of the 500 consistently DE genes had entropy of 2 (196 genes) or 3 (168 genes), while only a few genes were DE in only one macro-category (59 genes with entropy of 1) or had entropy of 4, 5 or 6 (60, 14 and 3 genes, respectively). The 59 genes with entropy of 1 were DE in the brain (31 genes) or in blood (28 genes) datasets, as these tissues are the most represented (Fig 2b and 2c).

**Fig 3:**
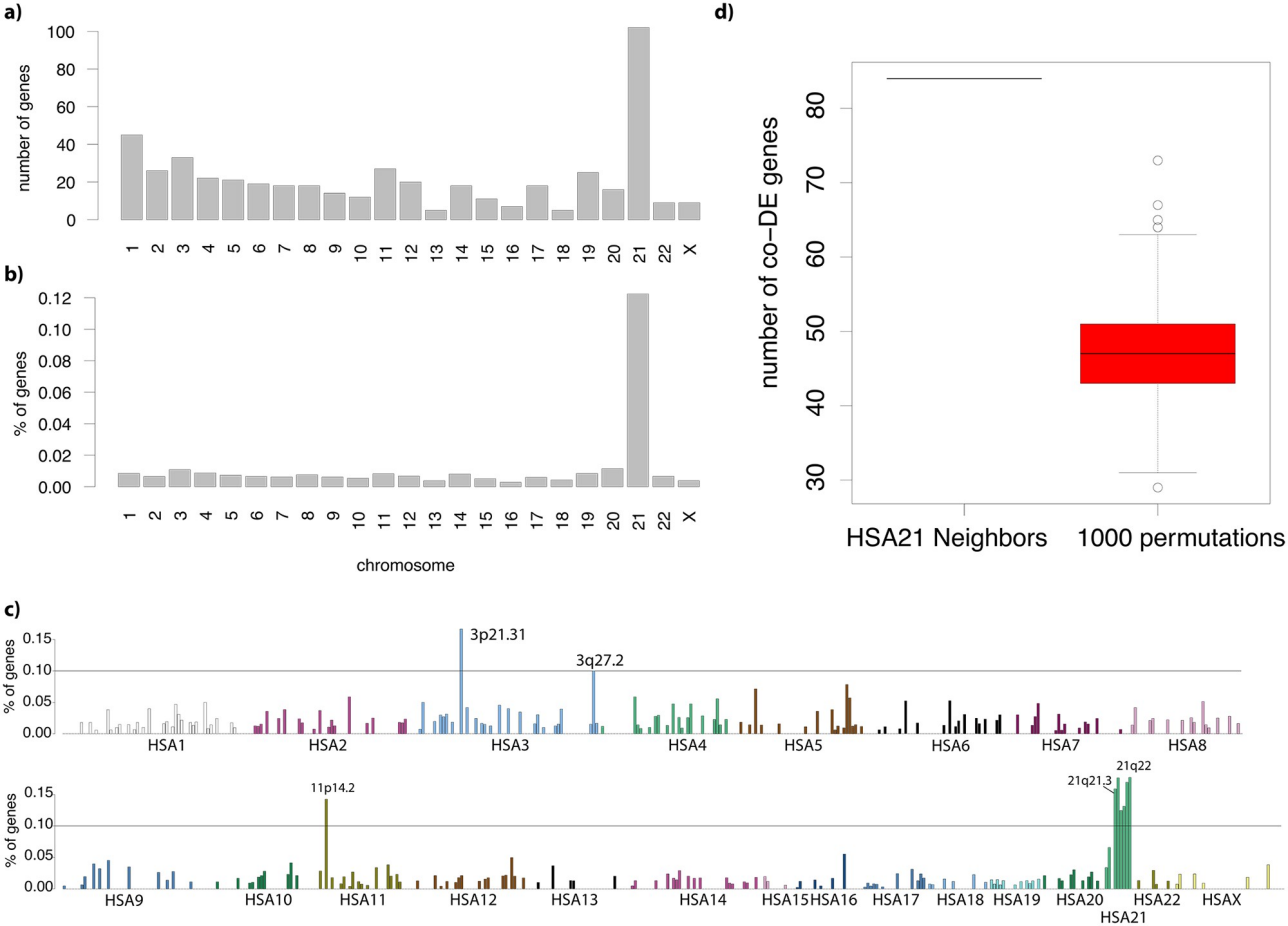
Most of the consistently DE genes map on HSA21. **a)** Barplot for the number of consistently DE genes per human chromosome. **b)** Same as in a) but normalized for the number of genes located in each chromosome. **c)** Same as in B but at the cytogenetic band resolution. **d)** Number of consistently DE genes that are HSA21 interactors (left). Boxplot of the number of consistently DE genes in 1000 random permutations (sampled with the same length as the total number of HSA21 interactors).

Even so, genes with entropy higher than 1, still could be preferentially DE in a tissue macro-category. To detect these genes preferentially DE in one tissue macro-category, we performed DE analysis but counting the occurrences of DE for each tissue macro-category: brain (55 comparisons); blood/bone marrow (27 comparisons); undifferentiated cells (12 comparisons); fibroblasts (9 comparisons); placenta/amniocytes (9 comparisons); and liver (2 comparisons). For each macro-category, we defined as preferentially DE genes those in the 5% right tail of the distribution of occurrences (see Methods). We detected in total 347 genes preferentially expressed in at least one tissue macro-categories (Fig 3b and 3c) of which 232 overlapped with the 500 consistently DE genes: 77 in brain, 84 in blood/bone marrow, 4 in fibroblasts, 7 in placenta/amnios, 42 in undifferentiated cells, and 18 that were consistently DE in more than one tissue macro-category (2 to 4 macro-categories). One example is *SOD1*, that we found preferentially DE in the brain, blood, fibroblasts and placenta/amnios. Of those, almost all (17/18 genes) mapped on HSA21 (Fig 2c). The remaining 268 genes were not found preferentially DE in any specific tissue macro-category.

The entropy level inversely correlated with the tissue specificity of the genes, with no tissue specific gene having entropy higher than 4 (Fig 2e). HSA21 genes showed higher entropy: 45% of the genes with entropy of 4 were located on HSA21, and this percentage rose to 80% for genes with entropy of 5, and 100% for genes with entropy of 6. Conversely, only 5% of genes with entropy of 1 were HSA21 genes (Fig 2d). The inverse correlation between number of tissue specific genes and entropy was even more evident when considering only HSA21 genes: 2 of the 3 HSA21 genes with entropy of 1 were tissue specific (see Methods), while 4/65 HSA21 genes with entropy of 3–4 were tissue specific (Fig 2f). This indicates that, as expected, HSA21 genes with lower entropy (e.g. found DE only in one tissue) were tissue specific while the ones broadly DE (with higher entropy) were expressed in many tissues.

### Most of the top-DE genes map on HSA21 genes and their interactors

Since DS is caused by trisomy of HSA21 we analyzed the chromosomal distribution of the 500 consistently DE genes. We found that 1 out of 5 mapped on HSA21 (102 genes; Fig 3a), corresponding to 12% of all HSA21 genes (Fig 3b). When zooming in to the band level (Fig 3c), we detected that the band with the highest number of consistently DE genes was the 21q21.3 and the complete 21q22. We then tested for the enrichment in genes among our consistently DE, that interact with HSA21 genes or proteins, as for example FOS (FBJ murine osteosarcoma viral oncogene homolog) that interacts with the HSA21 genes ETS2 and SUMO3.

To this aim, we retrieved highly stringent (>900) STRINGdb interactions and we found that 134 of the 500 consistently DE genes were HSA21 interactors, a number much higher than expected by chance (p-value <0.001, permutation test; Fig 3e).

### Genes with consistent upregulation are enriched in interferon-related categories and neutrophil activation

**Fig 4:**
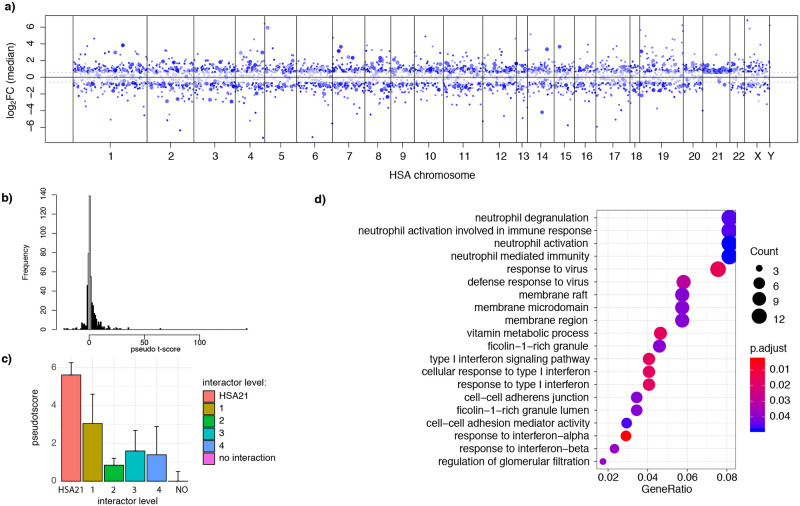
Genes with consistent upregulation are enriched in interferon-related categories and neutrophil activation. **a)** Manhattan plot where each dot is a gene deregulated in DS, the level of opacity indicates the pseudo t-score. The top 500 DE genes are plotted in bigger size. **b)** Distribution of pseudo-t-scores. The consistently up-regulated and down-regulated genes are in black. **c)** Barplot showing the average pseudo-t-score in HSA21 genes, their primary, secondary, tertiary interactors, and the other proteins of the network **d)** Gene ontology analysis for the genes with high absolute pseudo-t-score (in black in b).

As expected, most of the comparisons showed an upregulation of around 1.5-fold for HSA21 genes. However, some HSA21 genes were also down-regulated, as previously described [7]. We also detected a genome wide dysregulation including both down-regulated and up-regulated genes (Fig 4a).

To identify the genes showing the most consistent direction of change we computed a “pseudo t-score” on the 9727 genes DE in at least one trisomic versus euploid comparison (see Methods). This score was directly proportional to the mean fold change and number of comparisons in which a gene was found DE and inversely proportional to the standard deviation. We called “consistently up-regulated/down-regulated genes” those at the extremes of the distribution of pseudo-t-scores (see Methods and Fig 4b, black bars on the histogram, >1.96 and < -1.96 standard deviation). Concordantly, all the consistently up-regulated/down-regulated genes are included in the 500 consistently DE genes.

We found 147 up-regulated genes and 35 down-regulated genes among the consistently DE. None of the consistently down-regulated genes mapped on HSA21, even though some HSA21 genes were down-regulated in specific comparisons. We hypothesized that the consistently down-regulated genes could be targets of miRNA mapping on HSA21, since miRNA accelerates target mRNA degradation[8] and miRNAs are known to play a role in DS phenotype[9]. We found that this was the case for 4 genes, a number significantly higher than expected by chance (p-value for the Fisher Exact Test = 0.001044): COX8A (validated target of miR-802 [10]), TCF7L2 (validated target of miR-4759 [11]), SMOC1 and IGSF8 (validated targets of miR-6130 [10,12]). Other genes could be down-regulated because of specific deletions in mouse models. This is the case, for example, of ITGB8 and TMEM196 that map on a MMU12 deletion, characteristic of Ts1Cje mice. However, the scenario is more complicated as DNAH11, even if it maps in the same deleted region, is found upregulated in the same study[13]. Moreover, we also found ITGB8 and TMEM196 down-regulated in iPSCs derived from fibroblasts of trisomy 21 individuals, suggesting that other mechanisms might be responsible for the observed consistent down-regulation[14].

67/147 of the consistently up-regulated genes were on HSA21 (66% of the DE genes), and 80/147 mapped on other chromosomes. The genes with the highest pseudo-t-score were HSA21 genes, followed by their interactors. Moreover, second- and third-degree interactors had lower pseudo-t-scores and genes that did not interact at all had a pseudo-t-score around zero, meaning that direction of their change was inconsistent (Fig 4c). When exploring the enrichments for the set of consistently up-regulated/down-regulated genes (Fig 4d), we found enrichment in interferon-related categories (inflammatory genes up-regulated from x1.6 to x9) and neutrophil activation (up-regulated from x1.5 to x6.7).

### The DS network: Co-differentially expressed genes

**Fig 5:**
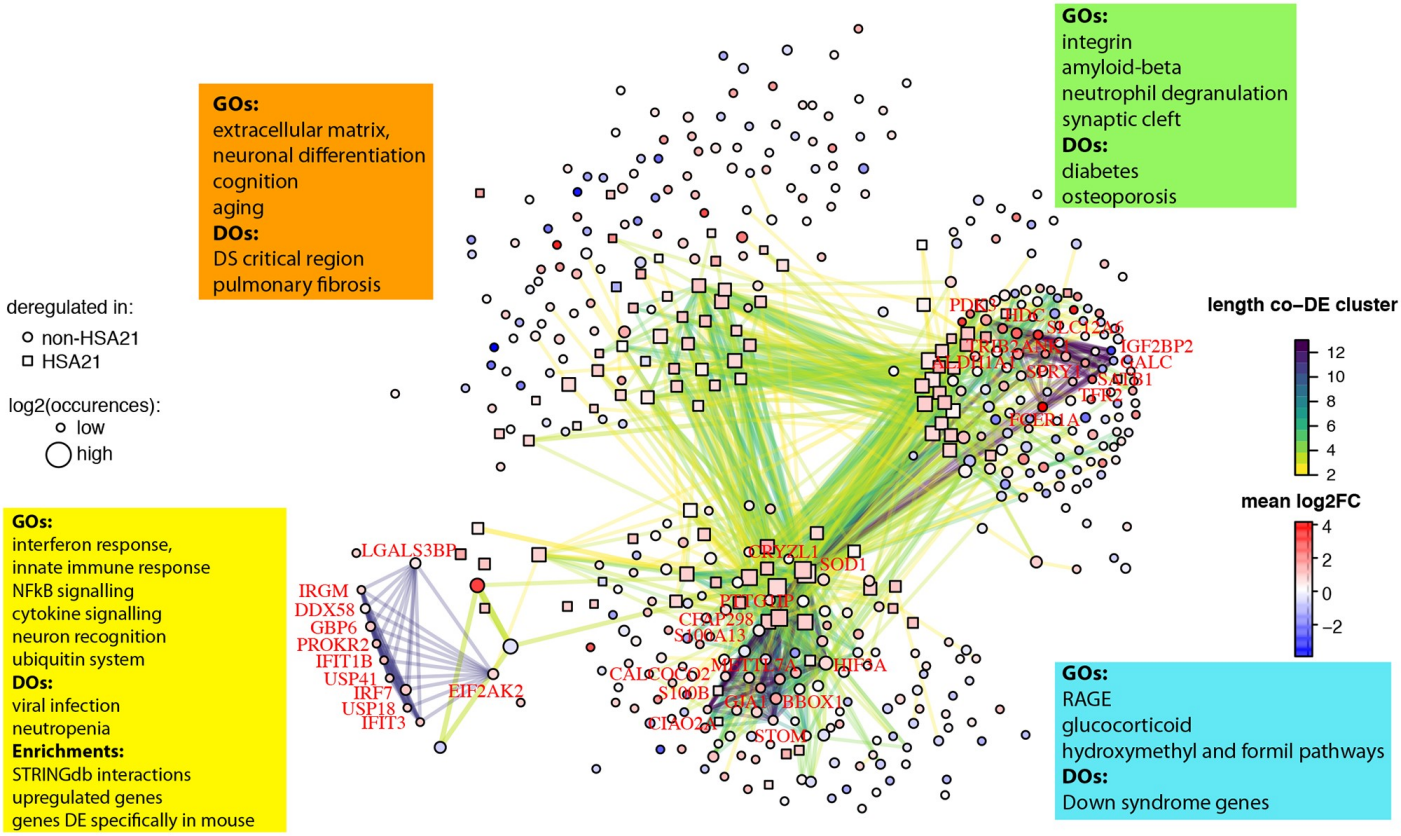
DS genes form a modular network. DS network where each node is a gene found DE in at least 4 comparisons. Edges represent co-DE events between genes colored based on the size of the co-DE cluster. Edge thickness is proportional to the number of occurrences of co-DE and the STRINGdb score. We did not plot the edges corresponding to co-DE pairs found in less than 4 comparisons). Size is proportional to the log_2_ of the occurrences of DE. Genes encoded by HSA21 are depicted with a square, non-HSA21 with a circle. The significant enrichments for each module are indicated with boxes of different colors.

We built a network in which each node is a gene found consistently DE, and each edge connects two genes that are co-differentially expressed (Fig 5). The thickness of the edge is proportional to the number of comparisons in which a given gene pair was found consistently co-DE. We also detected larger groups of genes co-DE in at least 4 comparisons (consistently co-DE gene clusters).

With this strategy, we detected two consistently co-DE clusters of 13 genes, both including the HSA21 *SOD1* gene. The first included: "ANK1”, “GALC", "PDK3", "TRIB2", "IGF2BP32", "TFR2", "SLC12A6", "HDC”, “SOD1", "SPRY1", "ALDH1A1", "FCER1A", and "SATB1", of which only SOD1 mapped on HSA21. All these 13 genes are co-DE in acute megakaryoblastic leukemia (AMKL) and acute myeloid leukemia (AML) mononuclear cells.

The second cluster included the HSA21 genes “SOD1”, “CFAP298”, “S100B”, “PTTG1IP”, and “CRYZL1”, together with the non-HSA21 genes “HIF3A”, “BBOX1”, “CALCOCO2”, “STOM”, “GJA1”, “CIAO2A”, “METTL7A”, and “S100A13”. These genes are co-DE in human postnatal cerebellar and primary visual cortices, and in adult dorsolateral prefrontal and inferior temporal cortices.

Besides the detection of these functionally significant clusters in our molecular network we performed a modular analysis to detect subgraphs of highly interconnected nodes. We identified four modules that we named green, blue, orange, and yellow. The consistently co-DE clusters guided the modular analysis. In fact, the 13 genes of the first cluster formed the green module, while the second cluster containing 13 genes (including SOD1) was part of the blue module. We also detected one module (orange) of genes interacting with the green and blue module and another module (yellow), containing a cluster of 11 genes. This cluster included the non-HSA21 genes "EIF2AK2", "PROKR2", "DDX58", "LGALS3BP", "IFIT3", "USP41", "GBP6", "USP18", "IRF7", "IFIT1B" and "IRGM" related to inflammation and ubiquitin systems. These 11 genes were found in the forebrain (E15.5) and cerebellum of Dp16 and Ts1Cje mouse models.

We then performed enrichment analysis to detect biological categories both specific and common in each of the modules. The green module was enriched in extracellular matrix (integrin), amyloid-beta, neutrophil degranulation, and synaptic cleft; the blue module in RAGE ("receptor for advanced glycation end products"), glucocorticoid, hydroxymethyl, formyl, and Down syndrome; the orange module extracellular matrix, negative regulation of neuronal differentiation, cognition, aging; and finally the yellow module contained many up-regulated genes enriched in interferon response, innate immune response, neuron recognition, ubiquitin, virus, NFkB signaling, and cytokine signaling.

### co-DE gene pairs are involved in common gene ontologies

Genes that are found co-DE could be also co-regulated, might interact, or be involved in the same biological pathway. For this reason, we analyzed the overlap of the connected nodes (gene pairs) of our network with the STRING databases (STRINGdb) of functional and physical interactions, and with the Gene Ontology (GO) database.

**Fig 6:**
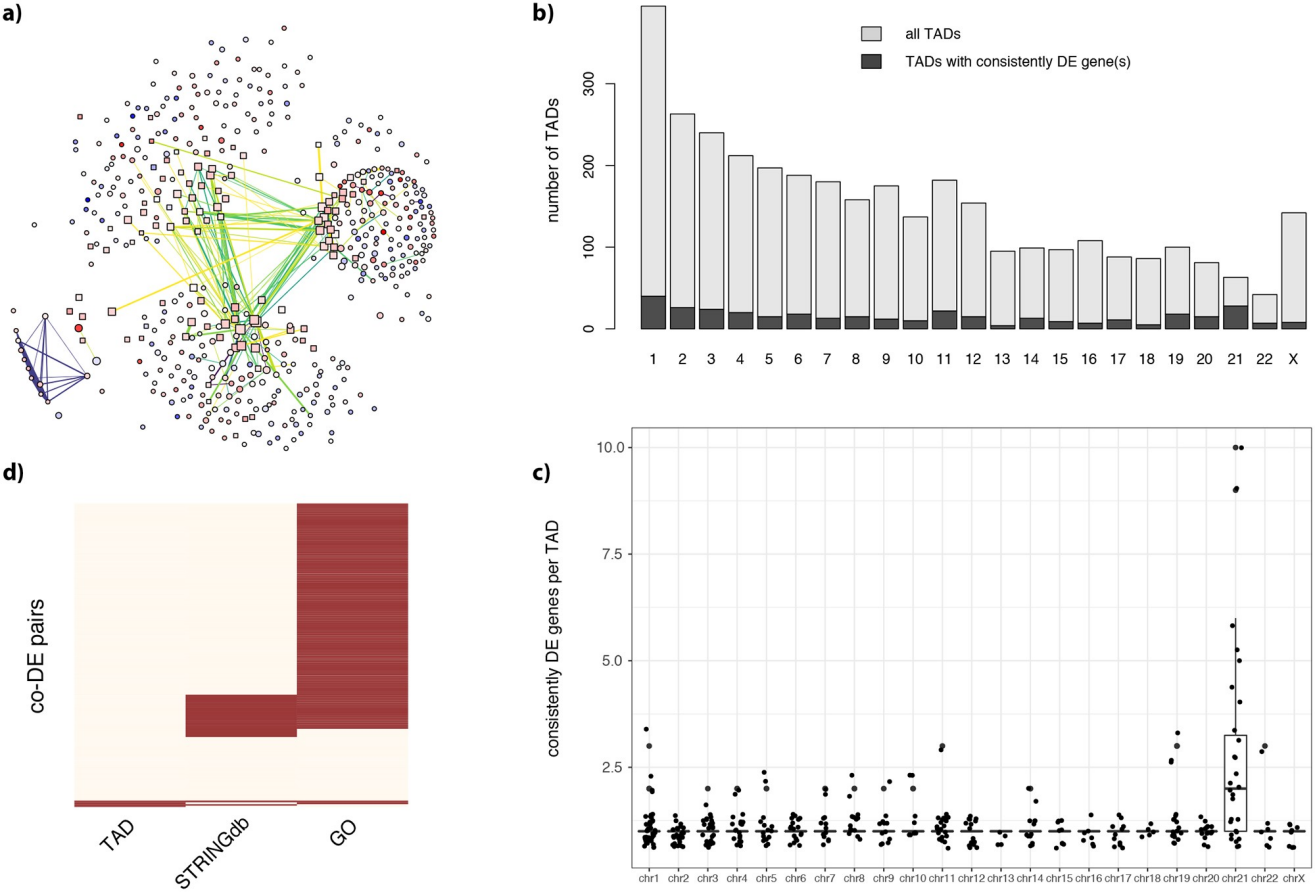
80% of the consistently co-DE pairs are involved in STRINGdb interaction, belong to the same GO or are located on the same TAD. **a)** DS co-DE network (as in Fig 4) where only the consistent co-DE edges with a STRINGdb interaction are reported **b)** Barplot representing the number of TADs per human chromosome. TADs containing at least one of the top-500 genes are in dark gray **c)** Boxplot showing the number of consistently DE genes per TADs per each chromosome **d)** Heatmap showing the 1243 edges consistently co-DE gene pairs that are in the same TAD, in the same Gene Ontology or involved in a STRINGdb interaction.

STRINGdb revealed 2377 interactions involving 262 of the 280 genes from our consistently co-DE gene pairs. However, only around 15% of the edges of our network represented an actual STRINGdb interaction (186 total interactions, Fig 6a). We found that the yellow module was the one with more of these co-DE gene pairs enriched in actual STRINGdb interaction (Fig 6a and 6d), indicating that gene co-expression might suit the physical and/or functional interaction between gene products.

We then checked if the number of co-DE gene pairs might reflect the membership to common GOs. In fact, 75% of the edges of our network (938 out of the 1243 co-DE gene pairs) was annotated in common GO categories (Fig 6d) which corresponds to 79% of the pairs with also a STRINGdb interaction. Therefore, 977 out of the 1243 genes pairs were involved in a STRINGdb interaction and/or in the same GO category (Fig 6d). Examples of co-DE pairs with the highest number of common GOs are SOD1 and PRDX1–belonging to categories related to antioxidant protective role in the cytoplasm of cells–and vinculin with gelsolin, involved in actin related-pathways–the first anchoring F-actin to the membrane and the second in assembly and disassembly of actin filaments. The GOs supported by more events of co-DE included oxidoreductase activity, neutrophil degranulation, positive regulation of transcription, defense response to virus and other immune system processes, but also cell projection, myelin sheath, neuronal cell body and metabolic process. This supports the idea that co-DE events might reveal biological processes affected in DS.

### The TAD structure does not explain most of the co-regulation events

Another mechanism explaining co-DE events could be the epigenetic deregulation of genes belonging to the same topologically associated domains (TADs). TADs are 3D regions of the genomes with genes co-regulated in blocks. We used the publicly available boundaries from Dixon et al. 2012 of human Embryonic Stem Cells (hESC), including 3127 domains, as these regions are conserved across cell types and regions. We mapped 434 of our 500 consistently DE genes on 355 TADSs (Fig 6b). 28 of these TADs mapped on HSA21 (out of the 35 TADs on this chromosome), and HSA21 was also the chromosome with more consistently DE genes in the same TADs (up to 10), probably because most of the consistently DE mapped on HSA21 (Fig 6c).

We then checked how many of the events of co-DE occurred between two genes located in the same TAD. Out of`181 of possible combinations given the 434 genes mapping onto TADs, only 27 corresponded to an actual event of co-DE between two genes, involving nine TADs: 1 on HSA7, 1 on HSA10, and 7 on HSA21 (one on band 21q21.1, two on 21q22.11, one on 21q2.12 and 3 on 21q22.3). We also found 6 groups of 3 co-DE genes that were on the same TADs. 9 of these genes mapped on two HSA21 TADs (one TAD on 21q22.1 and one on 21q22.3).

### DS and disease association

We interrogated the DisGeNet database that contains 1135045 gene-disease associations (GDAs) between 21671 genes and 30170 diseases, disorders, traits, or clinical or abnormal human phenotypes. Of our top 500 DE genes 467 were involved in 55477 GDAs, of which 454 were classified as “strong” or “definitive evidence level” in DisGeNet and 189 were associated with a given disease in at least 2 sources (and therefore more reliable). When looking at gene co-DE pairs, more than 87% of all gene pairs were involved in the same disease class, and 65% in the same disease.

**Fig 7:**
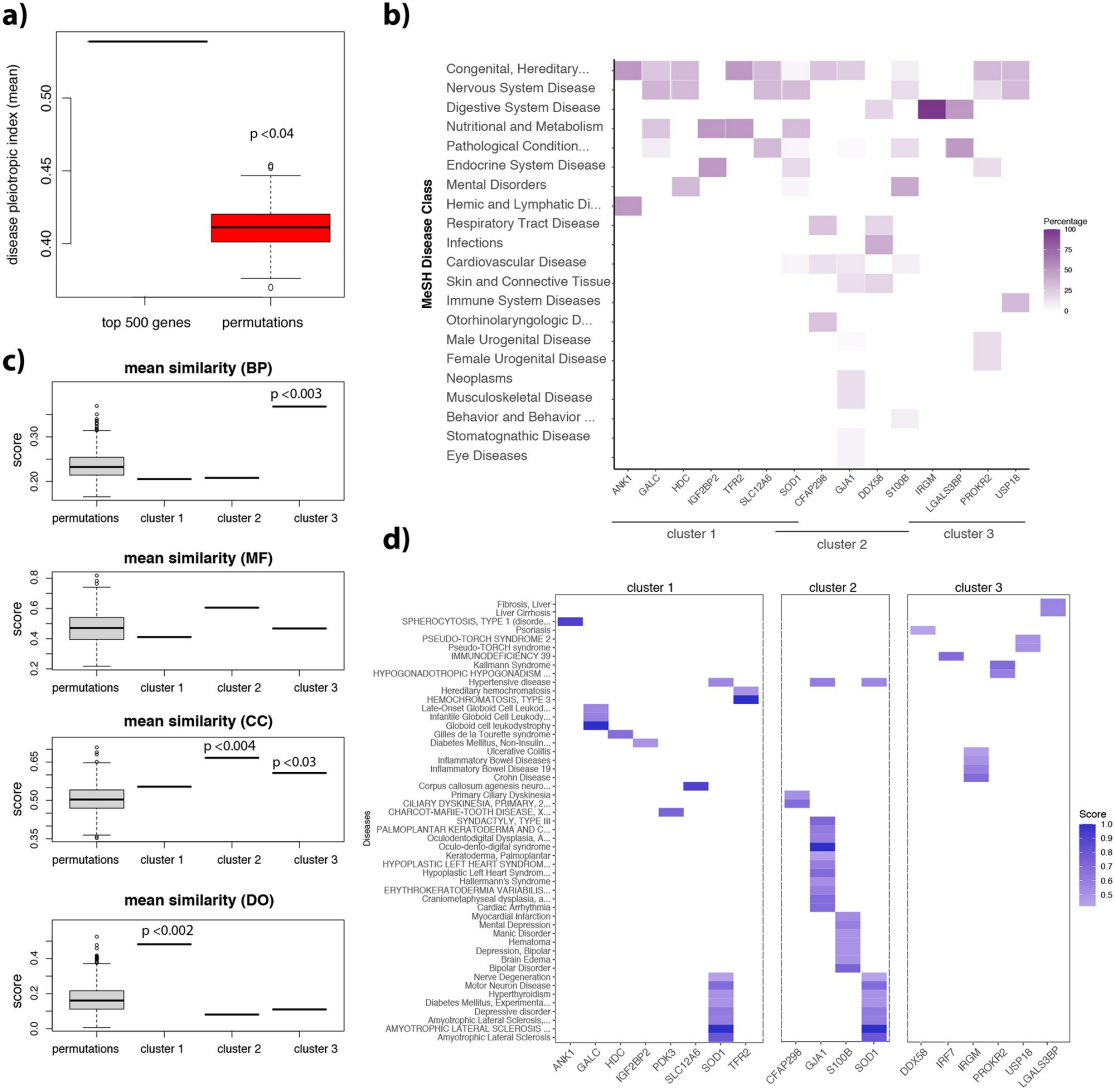
Disease association in DS genes. **a)** Boxplots comparing the mean disease pleiotropic indexes of 1000 permutation of 500 random genes compared to the mean disease pleiotropic index of the top 500 genes. **b)** Heatmap showing the percentage of disease classes of the genes belonging to the three clusters. Genes with high pleiotropic scores are associated with different disease classes. **c)** Boxplots comparing the mean similarity score of 1000 permutation of 12–13 random genes compared to the mean similarity score of the genes belonging to our three co-DE clusters. Similarity was assessed (from top to bottom panel) using the GOs biological processes (BP), molecular function (MF), cell component (CC) or disease ontology (DO). **d)** Heatmap showing the GDA’ scores of genes belonging to the three clusters.

We then considered the “disease pleiotropic index” (DPI) from DisGeNet, an index proportional to the number of the different MeSH disease classes of the diseases associated with a given gene. The mean DPI of the top 500 DE genes was higher than what expected by chance, indicating an enrichment for genes associated to diseases belonging to different classes (Fig 7a, p<0.04, permutation test with 500 gene lists randomly sampled from the list of 9727 genes found DE at least once). We found GDAs in all the three largest clusters of co-DE genes of our network, involving 8 genes in cluster 1 and 4 in cluster 2, and 6 in cluster 3 had GDAs detected in at least two sources (Fig 7b).

The genes belonging to the same cluster belonged to common gene ontology (GO) categories or disease ontology (DO). The structure of the GO and DO graph can be used to measure the functional similarity of the genes annotated with those ontologies by using the so-called semantic similarity analysis, a method that estimates the functional similarity based on the annotation statistics of the common ancestor terms[15] (see Methods). This analysis showed that the genes belonging to the same cluster had a higher semantic similarity score, meaning that they belonged to common and/or GO and DO (Fig 7c and 7d). Genes in cluster 1 showed a high semantic similarity using GO annotations, being enriched in the same disease ontologies (Fig 7c). Expectedly, SOD1 was the gene with the highest number of GDAs including nerve degeneration hyperthyroidism, hypertensive disease, diabetes mellitus and depressive disorders, which are known comorbidities in Down syndrome. Other interesting GDAs involved the IGF2BP2 genes and diabetes mellitus, and GALC and seizures. Cluster two also included the SOD1 gene but it also included other genes with high DPI such as GJA1, associated with cancer, hypertensive disease, heart anomalies and S100B, associated with schizophrenia, myocardial infarction, mental depression and bipolar disorders (Fig 7d). The genes in this cluster had common cellular components including dendritic and axonal proteins (Fig 7c). Finally, cluster 3 mainly included genes involved in inflammatory disorders including especially interferon-related biological processes (Fig 7c).

### The most consistent DE genes of our meta-analysis are found most significantly DE in an independent dataset

**Fig 8:**
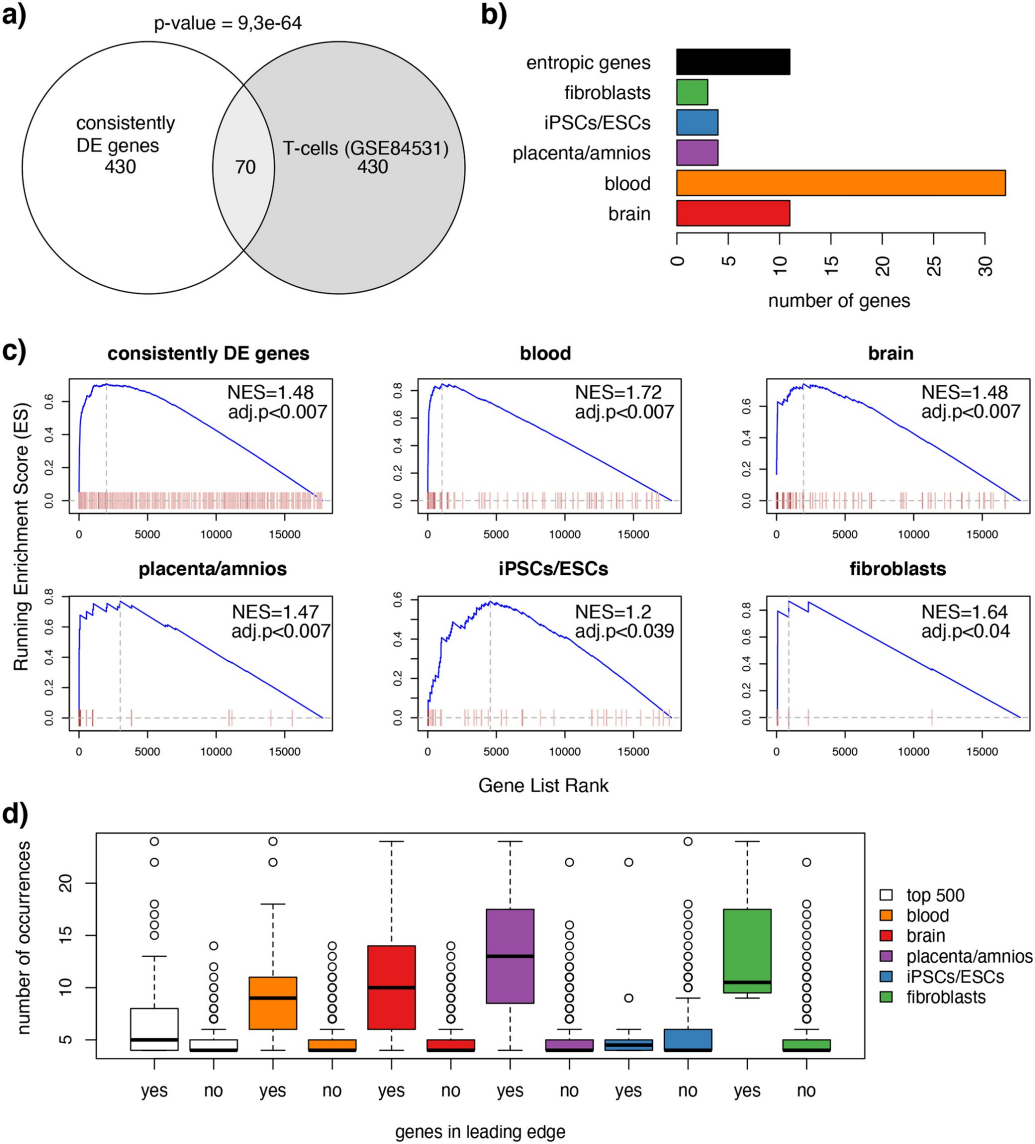
Genes DE in trisomic T-cells significantly overlap with our consistently DE genes, especially with genes consistently DE in blood. **a)** Venn Diagram showing the overlap between the consistently DE genes found in our meta-analysis and the top-DE genes in trisomic T-cells. The p-value of the fisher test for the enrichment is printed on top. **b)** Barplot showing the number of genes found DE in trisomic T-cells overlapping with the consistently DE genes in one or more tissue macro-category. **c)** Gene set enrichment for the top 500 consistently DE genes and the genes preferentially DE in each macro-categories across the list of the genes detected in trisomic T-cells ranked by their significance. Each red bar is a gene in the gene set, whose intensity of red is proportional to the number of occurrences. **d)** Boxplots showing the number of occurrences in the genes of the leading edge (left side of the distributions in panel c) compared to the ones not in the leading edge (right side).

Our meta-analysis led to the identification of genes that are consistently DE across different comparisons and in specific macro-categories. This means that in future studies on DS we would expect a significant part of the genes highlighted in this meta-analysis to be found DE. In order to check this, we separately analyzed another dataset that was not used in this meta-analysis, T-cells from disomic and trisomic individuals [16]. We therefore performed a DE analysis selecting the top-500 DE genes from this specific study. According to our expectation, we found a significant overlap of 70 genes (14%, Exact enrichment p-value from the Fisher Test = 9.288694e-63) between genes DE in T-cells from disomic and trisomic individuals and the consistently DE genes of our meta-analysis (Fig 8a). Expectedly, among the top-DE genes in trisomic T-cells we found 10 of the 18 genes that in our meta-analysis were found consistently DE in more than one tissue macro-category and 32/100 of the genes consistently DE in blood, accordingly with the origin of the sample of this comparison (Fig 8b). The analysis of the overlap however, was limited by the choice of our threshold for DE (adj-p-value <0.05 and absolute fold change >1.5). Therefore, we performed a classical gene set enrichment analysis (see Methods), using as gene sets all the top 500 consistently DE genes or the ones preferentially DE in each tissue macro-category, and ranking all genes in the independent T-cell dataset from the most to the less significantly changing. All the gene sets (both the consistent DE genes and the preferentially DE in the different tissue macro-categories) showed highly significant enrichment scores, meaning that they were significantly changing in the T-cell dataset, with the highest normalized enrichment score for the “blood” gene set (Fig 8c). Moreover, the genes with the highest running enrichment score (the enrichment score at the maximum point in the ranked list of genes) had the highest number of occurrences in our meta-analysis (the ones on the right of the dotted vertical lines). This means that the more a gene was consistently DE in our meta-analysis, the more it was likely to be highly significant in the T-cell comparison. This indicates that even if the T-cell sample was not used in our meta-analysis, its transcriptomic profile was partially predicted, being enriched in consistently DE genes, especially found in blood samples.

### Differences in gene coverage across platforms minimally affected the top-DE genes and the preferentially DE genes in tissue-macro-categories

Uneven platform distribution among tissue macro-categories might bias our entropy values and detection of preferentially DE genes in each macro-categories, influencing the list of consistently DE genes. For this reason, we investigated to what extent this was true.

We found a significant, though mild (S5A Fig, Pearson correlation = 0.16), correlation between the occurrences of DE expression versus the number of comparisons were a gene was detectable (S5a Fig). Specifically, genes DE in more than 5 contrasts were detected in at least 50 comparisons, while the ones detected in more than 15 contrasts were detectable in more than 80 comparisons. However, many genes detected in more than 80 contrasts were DE in only 1 comparison.

Given this nonlinear and low correlation, we could not normalize the occurrences of DE for the number of comparisons were a gene is detectable. However, we were able to estimate the expected “false negative rate” due to platform-specific coverage limitation. To this purpose we mapped the genes DE in comparisons coming from RNA-seq experiments (where there is potentially no coverage limitation) on all other platforms. Therefore, we could rank platforms both by their coverage and their average percentage of loss of DE genes due to coverage limitations (S5C Fig). We performed simulations adding randomly the expected proportion of DE genes to those platforms with coverage limitation (picking them from the set of genes not detectable in that specific platform), and recalculating the top-DE genes and preferentially DE genes. There was a significant and high correlation (0.93, p< 2.2e-16) between the ranking of DE occurrences from the simulations and the actual ranking, meaning that gene ranking only slightly changed (S5C Fig). We calculated the Szymkiewiz-Simpson coefficient (defined as the fraction of overlap over the smaller set) in the top-500 and preferentially DE genes for each macro-category. The mean overlap of the simulated top-500 genes with the actual one was higher than 95%, meaning that most of the consistently DE genes remained the same. The impact on macro-categories was even lower: on average no new preferentially DE gene in a given macro-category was found, with only maximum 10% of new genes appearing in some of the simulations (S5D Fig).

## Discussion

In the present study, we performed the most comprehensive meta-analysis of Down syndrome (DS) transcriptomic data. Meta-analysis of heterogeneous data has been successfully applied successfully to disentangle complex phenotypes in cancer [17]. To date only one meta-analysis was published for DS using real-time PCRs, proteomic and microarray dated more than a decade ago. Here we decided to integrate all available genome-wide transcriptomic studies coming both from microarray and RNA-seq experiments. We reasoned that genes consistently deregulated across different datasets are most likely to be key in biological processes affected in DS. However, given the heterogeneity of the available datasets both in terms of platform (different microarray and sequencing techniques), species (both homo sapiens and mus musculus), and sources (from peripheral blood to brain tissue), we did not integrate our sample with a classical co-expression analyses, as these methods are sensitive to outliers and require high-quality homogenous data, especially in terms of platform. To overcome this limitation, we developed a simpler method that consists in performing, per each dataset, a differential expression analysis with the optimal statistical methods suitable for each specific transcriptomic platform (e.g. moderated t-statistics for microarray and negative binomial for RNA-sequencing data). We then integrated the lists of DE genes of each comparison to create a DS co-DE network where edges represent that the two genes are co-DE (DE in the same comparison). We thereafter used different metrics to score genes and groups of genes: the number of comparisons in which they are found DE, how consistent and high is their fold change (using what we call pseudo t-score to normalize for the consistency of the change and the number of events of DE), and their entropy (number of different tissue macro-categories where a gene is found DE). The advantage of this method is that it allows identifying consistent changes both at the level of”tissue macro-categories” and across different tissues. The biological significance of our method of integration is supported by the fact that most of our co-DE events reflect GO membership or disease association.

Using this approach, we found a highly significant overlap with the “DS genes” found in Vilardell et al. but our analysis included 9 times more comparisons and 8 times more transcriptomic studies (S3 Fig) and revealed 421 not previously identified DS genes (S4 Fig, p-value < 2.2e-16, Fisher’s Exact Test for Count Data). However, Vilardell’s list of DE genes also includes 245 genes not consistently found DE in our analysis. This is probably due to the different methodology used. We considered the number of comparisons in which a given gene was found DE, while Vilardell emphasized other metrics, such as the fold change and included in their analysis 18 real time PCR on already previously known target genes, and protein studies while we focused only on genome-wide transcriptomic studies. Our original approach identifies new genes of interest for DS, correlating their co-differential expression with their pathogenic significance. In fact, our meta-analysis not only confirmed many well-known DS genes, but also detected genes previously not identified. Interestingly, some of our novel DS genes seem to be involved in hippocampal mediated learning processes (S6 Fig). One of these was ETNPPL (Ethanolamine-Phosphate Phospho-Lyase), a gene located on human chromosome 4. This gene was found upregulated in our meta-analysis but while wild type mice significantly upregulated this gene upon performing the hippocampal dependent “Novel Object Recognition” task (p-value = 0.03175, Wilcoxon rank sum exact test), this induction was not detected in the trisomic Ts65Dn mice (S6b Fig).

### HSA21 genes show higher entropy than non-HSA21 genes

As expected, the highest percentage of consistently DE genes (genes DE in at least four comparisons) was from HSA21. HSA21 genes also have higher entropy (the number of tissue macro-categories in which a gene is DE), so that the level of entropy was lower in non-HSA21 genes, increased for HSA21 neighbors and finally HSA21 genes had the highest entropy. We validated our approach in an independent transcriptomic study in trisomic T-cells where we were able to predict 70 of the top DE genes [16]. Most of these DE genes were enriched in the “entropic” genes detected in our meta-analysis and in the genes consistently DE in the “blood” macro-category. Moreover, the level of consistency (number of occurrences of DE) correlated with the level of significance in the T-cell study, with the “blood” gene set having the highest normalized enrichment score (Fig 8).

The consistency of the DE of HSA21 genes and their increased entropy, align with the dosage imbalance of HSA21 genes (the organicistic hypothesis) as it is generally assumed that DS genome-wide deregulation is due to triplication of transcription factors and epigenetic modifiers. In fact, we detected two levels of transcriptional dysregulation: some genes, mainly HSA21 genes, are consistently DE in more than a tissue macro-category, while other genes, with lower entropy are more specific for one tissue macro category. Our results suggest that the impact of the trisomy might diverge in each tissue having a specific gene set deregulation, even though the triplicated genes are the same. This may reflect deregulation of complete biological processes, as we found HSA21 neighbors to be enriched over chance level in our list of consistently DE genes.

### The consistent upregulated genes are enriched in interferon-related categories and neutrophil activation

By computing a “pseudo-t-score” we identified genes showing the most consistent changes (up or downregulation) across comparisons. As expected, most HSA21 genes were consistently up-regulated with a 1.5-fold change with just a few cases of downregulation in specific comparisons consistent to previous reports. 11% of the consistent events of downregulation were on non-HSA21 genes regulated by three HSA21-coded mi-RNA. However, unexpectedly, also the majority of non-HSA21 genes was upregulated. We speculate that this prevalence for upregulation may result in part from the upregulation of *HMGN1* gene (found DE in 11 of our comparisons and with an entropy of 5) that results in the increase of genes belonging to the low to middle range of expression, as previously proposed [5]. Gene ontology on the consistently upregulated genes (mainly HSA21 genes), revealed enrichments in several interferon-related and neutrophil activation categories, but not in other blood-related categories. This could explain the neuro-inflammation and higher levels of circulating cytokines in individuals with DS [18,19].

### co-DE genes interact or belong to common categories

In our study we focused on how genes were consistently found differentially expressed together. We detected pairs but also larger groups of co-DE genes, i.e. concertedly DE in at least 4 comparisons. Different reasons may account for this concerted modulation: the products of two co-DE genes could be involved in a functional and/or physical protein interaction, in a common molecular process, or map in the same topologically associated domain. In most cases, an event of co-DE between two genes underlaid the membership of the two genes to a common GO, suggesting that entire processes, and not just individual genes, could be deregulated in DS. This happened for categories such as oxidoreductase activity (e.g. SOD1 and PRDX1), neutrophil degranulation, positive regulation of transcription, defense response to virus and other immune system processes, but also cell projection (e.g. anchoring, assembly and disassembly of actin by vinculin and gelsolin), myelin sheath, neuronal cell body and metabolic processes.

Recent evidence suggests that gene co-expression is explained by the TAD structure. However, we could explain only a very low proportion of our co-DE events by the topological structure. This could be because we used as TAD borders the ones reported in Dixon et al. 2012 of human Embryonic Stem Cells (hESC)[20]. Even if TADs are conserved across conditions, cell types and even species, their borders could vary, and this is considered an additional layer of epigenetic regulation.

### High enrichment in associated diseases reveals key comorbidity molecular pathways

DS is characterized by a high number of comorbidities with different prevalence [21], that may be explained by common pathogenetic mechanisms. However, despite the efforts in the last years, studies revealing those pathways are scarce. We tested to what extent our DS disease network would reveal pathways relevant for several disorders using DisGeNet. Interestingly, most of the genes of the network were pathologically relevant, being involved in Gene-Disease Associations. Also, the “disease pleiotropic score” was higher than expected, indicating an enrichment for genes associated with diseases belonging to different classes. Our three largest co-differentially expressed clusters include gene-disease association with known DS’s comorbidities. Genes belonging to cluster 1 had a high similarity in terms of Disease Ontologies, and therefore their deregulation in “cluster” might strongly contribute to the phenotype. This cluster was especially interesting since, although it was formed by genes co-DE in AMKL and AML mononuclear cells, it was enriched for diseases of the nervous systems suggesting possible common molecular mechanisms. This is reinforced by the fact that cluster 1 belonged to a community of the DS network rich in extracellular matrix (integrin), amyloid-beta, neutrophil degranulation, and synaptic cleft categories. Similarly, cluster 2 included genes found co-DE in cortical areas but still contained genes involved in RAGE, glucocorticoid, hydroxymethyl and formyl metabolism. Similarly, to cluster 1, also in cluster 3 despite the brain tissue origin (forebrain and cerebellum from both the Dp16 and Ts1Cje mouse models) we found many up-regulated genes enriched in interferon response, innate immune response, neuron recognition, ubiquitin, virus, NFkB signaling, and cytokine signaling, suggesting neuro-inflammation.

This high enrichment in different classes of associated diseases reflects the complex DS phenotype showing not only intellectual disability but several comorbidities [21]. In fact, we detected a few genes associated with DS comorbidities. A well-known example is *SOD1* related to neuroinflammation, hyperthyroidism, hypertensive disease (associated also with *GJA1*), diabetes mellitus (associated also with *IGF2BP2*) and depressive disorders (associated also with *S100B*). Other GDAs associated with known DS comorbidities included *GALC* and seizures; *GJA1* and cancer, hypertensive disease, heart anomalies; *S100B*, associated with schizophrenia, myocardial infarction, mental depression and bipolar disorders; and *DDX58* and infection that would reflect the higher susceptibility to infection for DS patients [22]. Most genes of the three co-DE clusters were associated with the disease class “congenital and hereditary abnormalities”, nervous system disease, nutrition/metabolism or digestive system disease and cardiovascular disease, all belonging to DS symptoms and comorbidities [21]. Genes with higher pleiotropic scores in our analysis could thus potentially be interesting candidates to target not only the cognitive impairment but also co-morbidities, as they are associated with different diseases.

### Limitations

One caveat of our study is that the different tissue macro-categories are not equally represented with most of the samples coming from blood or brain. Therefore, it is less likely to find bona fide tissue specific genes that do not belong to these two macro-categories (since for many tissues we do not have enough datasets for the integration).

Another limitation is related to species. We had to map mouse genes on their human orthologous and not always there is a 1:1 correspondence. When looking at the species, the contribution of human studies to our meta-analysis was higher (8671 out of the 9511 genes DE at least in one dataset) than the mouse (1720/9511), with just 9% genes in common (880). However, we found that in our network 55.6% of the genes are exclusively found in human and only 2.6% of the nodes are exclusively found in mouse (enriched in the yellow cluster), while 41.8% of the genes are found in both mouse and human (almost 5x than what expected, 3-sample test for equality of proportions, p = 1.16e-146). This is probably because genes that are both found in *homo sapiens* and *mus musculus* have a higher chance to be selected as “consistent DE genes” and therefore be included in the top 500 genes.

Last but not least, even if we limited our analysis to genome-wide approaches, genome coverage was far from being 100%. For RNA-seq experiments, it depended on sequencing depth, while for microarray experiments, on the platform used that might, for example, have probes only for protein coding genes. We actually showed that the platforms with lower transcriptomic coverage were the ones showing a higher predicted rate of false negative DE gene (the correlation was not 1 since genes are not randomly chosen on platforms). While the false positive rate and false negative rate depended on the sample size and variability of each comparison, this “loss” in positives (DE genes) is exclusively platform dependent. This means that our analysis is less powerful in detecting lowly expressed genes or genes not present on certain arrays (e.g. genes for non-coding RNAs). However, these genes have often unknown or unsure function, and are therefore not annotated in pathways or gene ontologies. Fortunately, our lists of consistently DE genes and preferentially DE genes in each micro-category were only modestly affected by this bias, as shown by our simulations (S5 Fig). We therefore concluded that, while it is true that the absence of certain genes in some of the platform represents a bias, with some genes being less likely to be called DE due to coverage limitations in some of the platforms, this bias is mild due to the specific configuration of our meta-analysis. We think however that the impact of gene coverage should be assessed in future meta-analyses integrating platforms others than RNA-sequencing.

## Conclusion

Our study reveals that the genome wide deregulation produced as a consequence of trisomy 21 is not arbitrary, but involves deregulation of whole molecular cascades in which both HSA21 genes and HSA21 interactors are more consistently deregulated compared to other genes. In fact, we found that gene deregulation happens in “clusters”, meaning that groups of genes are found consistently deregulated. These genes often belong to the same GO category, and are associated with the same disease class. Genes with the most consistent changes were enriched in interferon related categories and neutrophil activation, reinforcing the concept that DS is an inflammatory disease.

Known genes, such as SOD1, and new ones whose importance has been highlighted by this meta-analysis (such as *ETNPPL*), could be extremely used by the scientific community working on DS both for generating/confirming hypotheses and to support new putative therapeutic candidates. Moreover, the consultation of our results can be easily done through our web-application application while our method will be available to other kinds of transcriptomic datasets thanks to the development of the R package “metaDEA” (https://github.com/Ilarius/metaDEA.).

## Methods

### Data sources

Raw data were extracted from Gene Omnibus Expression GEO [23], and Array Express [24]. S1 Table lists all datasets that were used in this analysis, with their PubMed and/or repository references.

### Analysis of microarray data

Regarding the analysis of microarray data, quality check, background correction, and normalization were performed using different R packages, because of the different platforms used:

- “Affy” [25] and “oligo” [26] were used for the analysis of Affymetrix GeneChip data at the probe level
- “Beadarray” [27] was used for illumina bead-based arrays

In most cases the differential expression analysis of microarray data was performed using moderated t-statistics [28,29] with the package limma computing the comparison “trisomic-disomic”.

When subsetting the samples in different comparisons was not possible (for example because of the limited sample size), we used the *duplicateCorrelation* function from the *statmod* package [30], blocking for other variables such as treatment, sex and age.

### Analysis of RNA-seq data

All RNA-sequencing experiments used in this meta-analysis were performed with the Illumina platform. Reads were downloaded from the *Sequence Read Archive* using *fastq-dump* and mapped to the *mm10* genome for mouse and to the *GRCh38*.*p12* genome for human samples using STAR with the recommended parameters according to each dataset [31]. For gene annotation of the mapped reads, we used *gencode*.*vM17* for mouse and *gencode*.*v28* for human samples. Differential expression analysis between trisomic and euploid samples was performed with *DESeq2* [32], blocking for external factors (sex, age, etc.) whenever possible.

### Gene annotation

For the annotation of the ensembl gene identifiers (IDs) we used the *biomaRt* package [33,34], using the *getLDS* function to map mouse ensembl gene IDs to their human orthologs; the *org*.*Mm*.*eg*.*db* and *org*.*Hs*.*eg*.*db* packages. For the annotation of the microarray probes we used the following R packages:

- Affymetrix Human Genome U133 Set: hgu133a.db
- Affymetrix Human Genome U133 Plus 2.0 Array: hgu133plus2
- Affymetrix Murine Genome U74v2: mgu74av2.db
- Affymetrix huex10 annotation data: huex10sttranscriptcluster.db
- Affymetrix hugene10 annotation data: hugene10sttranscriptcluster.db
- Affymetrix hugene20 annotation data: hugene20sttranscriptcluster.db
- Affymetrix mogene10 annotation data: mogene10sttranscriptcluster.db
- Affymetrix Mouse Genome 430 2.0 Array annotation data: mouse4302.db
- Illumina HumanWG6v2 annotation data: illuminaHumanv2.db
- Illumina MouseWG6v2 annotation data: illuminaMousev2.db
- Illumina HumanHT12v4 annotation data: illuminaHumanv4.db
- Illumina HumanHT12v3 annotation data: illuminaHumanv3.db

### Detection of consistently DE genes

In order to detect the most consistently DE genes we plotted the distribution of occurrences of DE expression for all genes in all comparisons (for detecting consistently DE genes across tissues) or in a given tissue macro-category (macro-category specific consistently DE genes). These distributions were heavy tailed with many genes being DE in only one comparison and fewer genes being DE in more comparisons. We defined as consistently DE those genes falling in the 5% tail of this distribution, being the probability to find by chance a gene with a higher number of occurrences of DE lower than 0.05. Analysis of the heavy tail distribution of gene occurrences was performed with the *poweRlaw* package [35]. This package allowed us to choose the best model for p-value computation choosing between log normal, exponential, poisson, or power-law by plotting the cumulative distribution function and comparing it with the empirical distribution. Of course, with this approach the confidence in detecting macro-category specific consistently DE genes was dependent on the number of available comparisons. The more the comparisons, the more the resolution. In the case of the liver for example, since there were only two comparisons, instead of a real distribution of occurrences the distribution of occurrences we had only two bars in the histogram: a lot of genes DE in only one of the comparisons, but few genes DE in both (low resolution of the distribution of occurrences).

### Calculation of pseudo-t-score

For each gene we calculated the mean log_2_FC across comparisons divided by the across-comparisons mean standard deviation over the square root of the number of comparisons. Only the comparisons where the gene is found DE are used in the computation of the mean value of that given gene. We then plotted the distribution of pseudo-t-scores that had very long tails and defined as consistently changing genes, the ones being in the 5% left (consistently down-regulated) and the 5% right (consistently up-regulated).

$${pseudotscore}_{i}=\frac{mean({{log}_{2}FC}_{i,j=1}^{i,n})}{sd({{log}_{2}FC}_{i,j=1}^{i,n})*\frac{1}{\sqrt{n}}}$$

where *n* is the number of comparisons in which the gene *i* is found DE.

### Tissue specific genes

Tissue specific genes were detected using the *TissueEnrich* R package [36] processing RNA-Seq data across 35 human tissues from the Human Protein Atlas (HPA) [37].

We used *TissueEnrich* default parameters based on the algorithm from the HPA [37]: genes with an expression level greater than 1 TPM (Transcripts Per kilobase Million) or FPKM (Fragments Per Kilobase Million) that also have at least five-fold higher expression levels in a particular tissue (or group of tissues) compared to the average levels in all other tissues were defined as tissue specific. Definitions, statistics and parameters used in this study are summarized in S2 Table and the scheme of the results in S1 Fig.

### Gene set enrichment analysis

Gene set enrichment analysis was used to investigate enrichment of consistently DE genes and preferentially DE genes for each macro-category (used as gene sets) along the list of genes detected in the T-cell dataset from the GSE84531 study ranked for the p-value of the trisomic-euploid comparison. Enrichment scores, Normalized Enrichment Score (NES), p-values for the enrichment and genes in the leading edge, were calculated as described in [35]. In brief, GSEA calculates the ES by walking down the ranked list of genes, increasing a running-sum statistic when a gene is in the gene set and decreasing it when it is not. The ES is the maximum deviation from zero encountered in walking the list. For a positive ES, the leading-edge subset is the set of members that appear in the ranked list prior to the peak score. In order to calculate nominal p-values for the enrichments and to the NESs we permuted the ranked list a 1000 times. The NES is calculated as the ES/mean(all permuted ES), while the p-values as (sum(permuted ESs>ES)+1)/(number of permutations +1), and corrected with the Benjamini-Hochberg correction.

### Other analyses

Enrichment analyses were performed with *clusterProfiler* [38]. Graph-based analysis and visualization was performed with the *igraph* package [39]. Modular analysis detected 4 modules using the function *cluster_fast_greedy*, that implements the fast greedy modularity optimization algorithm for finding community structure [40]. As a non-parametric test for assessing differences we used a permutation test with 1000 permutations. To avoid p-values of zero we added a pseudo count of “1” both at the numerator and denominator when assessing the fraction of permutation higher than our observed value. Other visualization tools used included *ggplot2* [41], *eulerr*, and the *tagcloud* package.

The association between gene and diseases was performed with the *disgenet2r* package querying the DisGeNet database [42].

The pairwise semantic similarity among GO terms and our list of genes was calculated with the *GOSemSim* R package [43,44]. The topology of the GO graph structure was used to compute semantic similarity according to Wang’s method using the "BMA" (best-match average) method for combining semantic similarity scores of multiple GO terms associated with protein or multiple proteins associated with protein clusters [15].

The significance of the overlaps was calculated with the Fisher Exact Test.

The micro RNA databases were queried using the *multiMiR* R package [45].

### Experimental validation

Experimental validation was carried out for the genes found consistently DE in the hippocampus whose importance, to our knowledge, was not previously described. To this aim we tested the expression levels in control conditions and upon exposure to a hippocampal dependent learning test.

Mice were generated by repeated backcrossing of Ts65Dn females to F1 hybrid males from C57BL/6IEiJ females and C3H/HeSnJ males (B6EiC3SnF1/J). The parental generation was obtained from the research colony of Jackson Laboratory (Bar Harbor, USA). Euploid littermates of Ts65Dn mice served as controls. Behavioral tasks and RT-qPCR have been conducted using 2 month-old mice.

#### Novel Object Recognition (NOR) test

For the Novel Object Recognition test in an initial habituation phase, each animal is allowed to freely explore an empty arena (45cm x 45cm) for 10 minutes. Each animal was returned to the arena after 24h and allowed to explore two identical objects made with plastic building blocks for 15 minutes. After a retention interval of 24h, the test was performed. In this 5 minutes phase, one of the two identical objects is replaced by a novel object of similar size but different shape. We measured memory recognition measuring the time passed exploring the novel over the total time passing exploring whether the novel or familiar object (Discrimination Index) Exploration time was considered when the animal’s head was looking at the object in a 2 cm range.

#### Hippocampi isolation, RNA extraction and cDNA synthesis

One hour after completing the test phase of the NOR task, the hippocampal tissues were dissected, snap-frozen in liquid nitrogen and kept at -80°C until processed. Prior to RNA extraction, hippocampi samples were homogenized by mechanical trituration with a pestle. Total RNA extraction was performed using the Qiagen RNeasy Mini Kit according to manufacturer’s instructions. RNA purity was assessed using a NanoDrop 2100 (Thermo Scientific). All RNA samples were subjected to DNase digestion with DNase I (Sigma). Single-stranded cDNA was synthesized from 1000 ng of total RNA using SuperScrip III Reverse Transcriptase and oligo(dT) primers (Thermo Scientific) following the manufacturer’s instructions.

#### Quantitative real-time PCR

All samples were run in triplicates. Briefly, RT-qPCR reactions were carried out using LightCycler 480 SYBR Green I Master Mix (Roche) on a LightCycler Real-Time PCR System (Roche). The final volume for each reaction was 20 μl with 500 nM of corresponding gene specific primers (Xpo7 forward primer: AGCAAAATGGCGGATCATGTG; Xpo7 reverse primer: GTGGCAAGGGGTTGTTTGTC; Etnppl forward primer: TAGTGACCTCCGATACCCAG; Etnppl reverse primer: CGTGCCAGACGTAAGGCTAA), and 5 μl of total cDNA diluted 1:5. A negative water control was included in each run. The thermal cycling was initiated at 95°C for 10 minutes followed by 45 cycles of 10 s at 95°C and 15 s at 55°C, the optimal annealing temperature for our target genes. Melting curve analyses were carried out at the end of each run of qPCR to assess the production of single, specific products.

## Supporting information

S1 Fig Summary of the analyses performed. We performed differential expression (DE) analysis on 127 comparisons coming from 67 studies. 92 of these comparisons (coming from 51 studies) had genes DE with at least 1.5 absolute fold change with a Benjamini-corrected p-value < 0.05. We first plotted the distribution of occurrences of DE per each gene both pulling all the data together or pulling them according to previously defined tissue macro-categories (see main text). This allowed us to define four groups of genes connected to DS: for the detection of genes consistently up-regulated/down-regulated (light blue) we calculated the pseudo t-score for all the 9511 genes (see Methods), we then plotted their distribution and took the 5% of both sides; we then calculated the consistently DE genes, disregarding tissue macro-categories (yellow) by taking the genes falling in the 5% right tail of the distribution of occurrences of the 9551 DE genes; we next performed the same analysis, but separating the data in tissue macro-categories. This allowed us to detect consistently DE genes in more tissue macro-categories (red), or only one tissue macro-category (green).
(PDF)

S2 Fig Heatmap showing the tissue distribution of the top-500 DE genes. Each row is one of the 500 consistently DE genes and each column a tissue type (**a**) or macro-category (**b**).
(PDF)

S3 Fig Our analysis is the most comprehensive meta-analysis for DS so far. **a)** Tag cloud, where the size GEO/arrayExpress ID is proportional to the number of comparisons comparing trisomic versus disomic samples included in the study (e.g. different tissues, conditions, age, models, etc.). In red are the datasets that were included in the Vilardell et al. paper. **b)** As in A but with all the comparisons shown.
(PDF)

S4 Fig Overlap with previous meta-analysis. Venn Diagram showing the significant overlap of our consistently DE genes with the list of DS genes from Vilardell[7].
(PDF)

S5 Fig The bias due to genomic coverage only mildly affect our meta-analysis. **a)** Plot showing the number a gene is found DE in our comparisons versus the number of times a gene is detectable. Each circle is one of the 9727 genes found DE in at least a comparison. **b)** Barplots showing the percentage of detectable DE genes in each platform (left) and the mean percentage of DE genes that would not be called “DE” due to coverage limitation in each platform. The error bars represent standard devation as this values were calculated from all the comparisons coming from RNA-seq experiments. The gradient of grays is proportional to the number of comparisons per each platform. **c)** Plot showing the positive correlation as indicated by the dashed red line between the actual rank of DE gene occurrences (x-axis) versus the mean rankings in the 1000 simulations. **d)** Boxplots showing the distributions of the Szymkiewicz-Simpson coefficients (overlap over the length of the smaller set) between the consistently DE genes and the preferentially DE genes in each macro-category with each of the respective simulated values from the 1000 permutations.
(PDF)

S6 Fig Our meta-analysis detects new DS candidates. Quantitative PCR (qPCR) analysis of candidate genes on total RNA extracted from whole hippocampi (N = 4–5 per group) from mice sacrificed after conducting the Novel Object Recognition memory task (NOR) or not (Naive). Gene expression values are normalized to GAPDH. Each individual mouse is represented by a dot in the plot. Bars show the mean value of Efficiency^(-ΔCt) for each group. **a)** A decreasing tendency in Xpo7 expression levels in the Ts65Dn hippocampus was observed (p-value = 0.1111, Wilcoxon rank sum exact test) **b)** The NOR task triggers the expression of Etnppl in the WT but not in the Ts65Dn hippocampus (p-value = 0.03175, Wilcoxon rank sum exact test).
(PDF)

S1 File Comma-delimited file with the information on the top-500 genes coming out from the meta-analysis: Ensemble gene ID, HGNC symbol, extendend name, chromosome band, occurrences of DE, mean and median log2 fold change (and its standard deviation); expression in the tissue categories, entropy (both for macro and micro tissue categories), pseudo-t-score, interactor level and TAD number. (CSV)

S2 File Comma-delimited file with the output when querying the DysGeNet database with the top-500 genes. (CSV)

S3 File R markdown file with the code for the differential expression analyses. Download the repository’s folder for running it.
(RMD)

S4 File R markdown file with the code for generating all the analyses and figures of this meta-analysis. Download the repository’s folder for running it.
(RMD)

S1 Table Information on the dataset used in this meta-analysis. (DOCX)

S2 Table Terms used in the meta-analysis. (DOCX)

## Abbreviations

AMKL: Acute megakaryoblastic leukemia
AML: Acute myeloid leukemia
BMA: Best-match average
BP: Biological process
CC: Cell component
DE: Differential expression/differentially expressed
DO: Disease ontology
DPI: Disease pleiotropic index
DS: Down syndrome
FOS: FBJ murine osteosarcoma viral oncogene homolog
FPKM: Fragments per Kilobase million
GDA: Gene-disease association
GO: Gene ontology
hESC: human Embryonic Stem Cell
HPA: Human protein atlas
HSA21: Human chromosome 21
ID: Identifier
MF: Molecular function
RAGE: receptor for advanced glycation end products
STRINGdb: STRING database
TAD: Topologically associated domain
TPM: Transcripts per kilobase million
