# Supplementary material for: Meta-analysis of transcriptomic data reveals clusters of consistently deregulated gene and disease ontologies in Down syndrome
Source: PLoS Comput Biol. 2021 Sep 27;17(9):e1009317. doi: 10.1371/journal.pcbi.1009317 (PMC8496798; doi:10.1371/journal.pcbi.1009317)
Supplement: S1 Fig — We performed differential expression (DE) analysis on 127 comparisons coming from 67 studies. 92 of these comparisons (coming from 51 studies) had genes DE with at least 1.5 absolute fold change with a Benjamini-corrected p-value < 0.05. We first plotted the distribution of occurrences of DE per each gene both pulling all the data together or pulling them according to previously defined tissue macro-categories (see main text). This allowed us to define four groups of genes connected to DS: for the detection of genes consistently up-regulated/down-regulated (light blue) we calculated the pseudo t-score for all the 9511 genes (see Methods), we then plotted their distribution and took the 5% of both sides; we then calculated the consistently DE genes, disregarding tissue macro-categories (yellow) by taking the genes falling in the 5% right tail of the distribution of occurrences of the 9551 DE genes; we next performed the same analysis, but separating the data in tissue macro-categories. This allowed us to detect consistently DE genes in more tissue macro-categories (red), or only one tissue macro-category (green). (PDF) [file pcbi.1009317.s001.pdf]

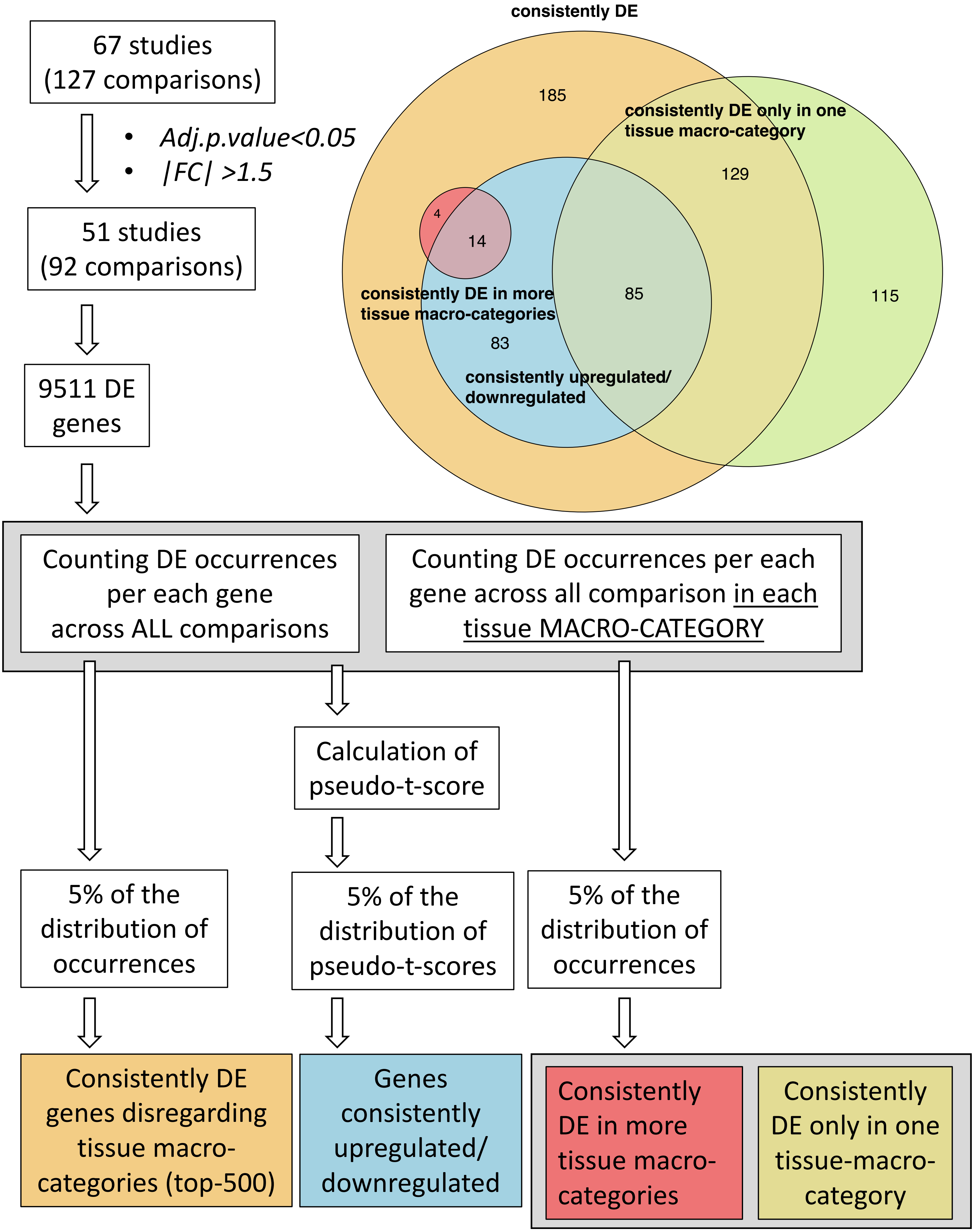
67 studies
(127 comparisons)
Adj.p.value<0.05
|FC| >1.5
51 studies
(92 comparisons)
9511 DE genes
consistently DE
185
consistently DE only in one tissue macro-category
129
4
14
consistently DE in more tissue macro-categories
85
115
83
consistently upregulated/ downregulated
Counting DE occurrences per each gene across ALL comparisons
Counting DE occurrences per each gene across all comparison in each tissue MACRO-CATEGORY
Calculation of pseudo-t-score
5% of the distribution of occurrences
5% of the distribution of pseudo-t-scores
5% of the distribution of occurrences
Consistently DE genes disregarding tissue macro-categories (top-500)
Genes consistently upregulated/ downregulated
Consistently DE in more tissue macro-categories
Consistently DE only in one tissue-macro-category
