# Supplementary figures and images for: Meta-analysis of transcriptomic data reveals clusters of consistently deregulated gene and disease ontologies in Down syndrome
Source: PLoS Comput Biol. 2021 Sep 27;17(9):e1009317. doi: 10.1371/journal.pcbi.1009317 (PMC8496798; doi:10.1371/journal.pcbi.1009317)

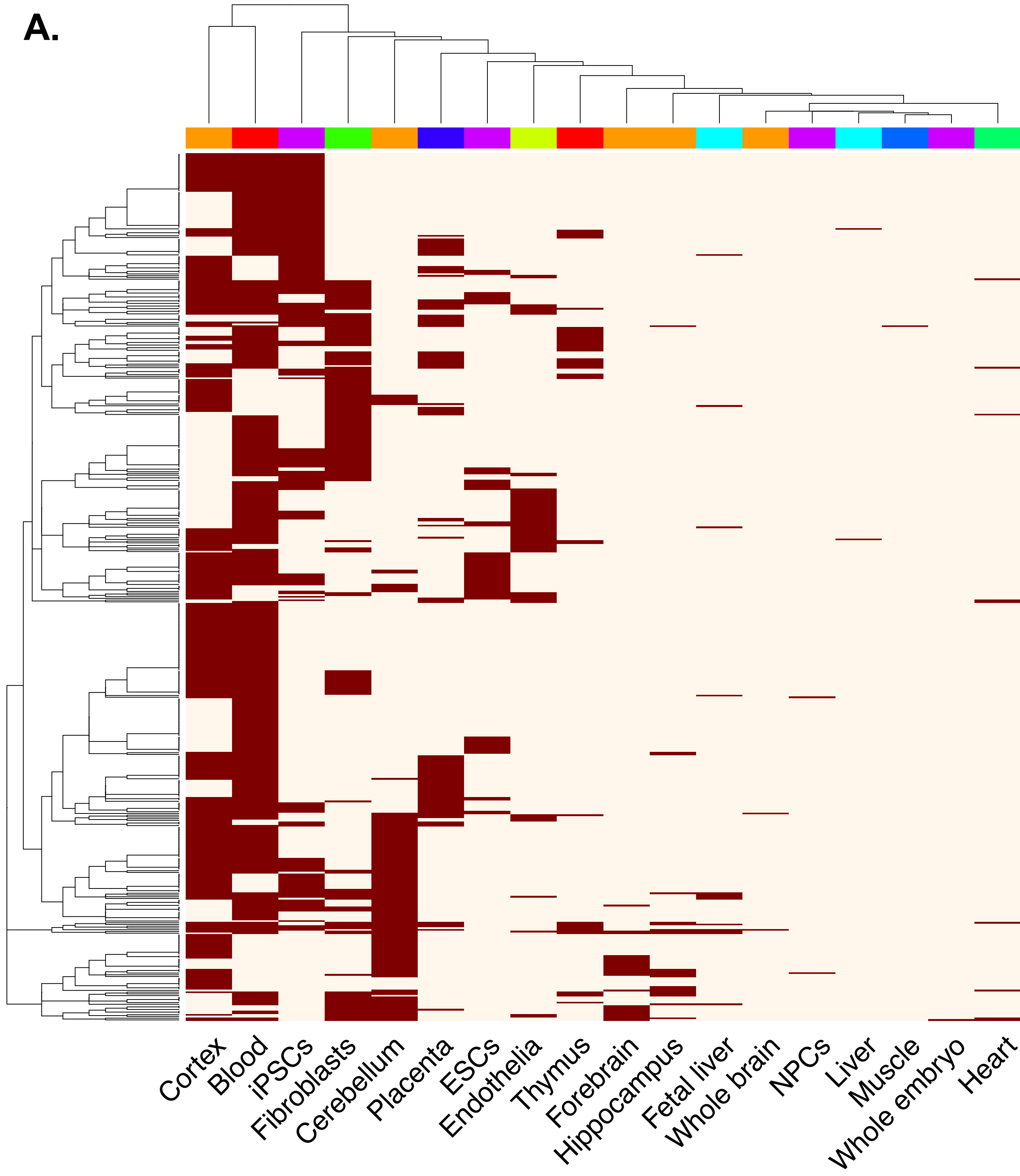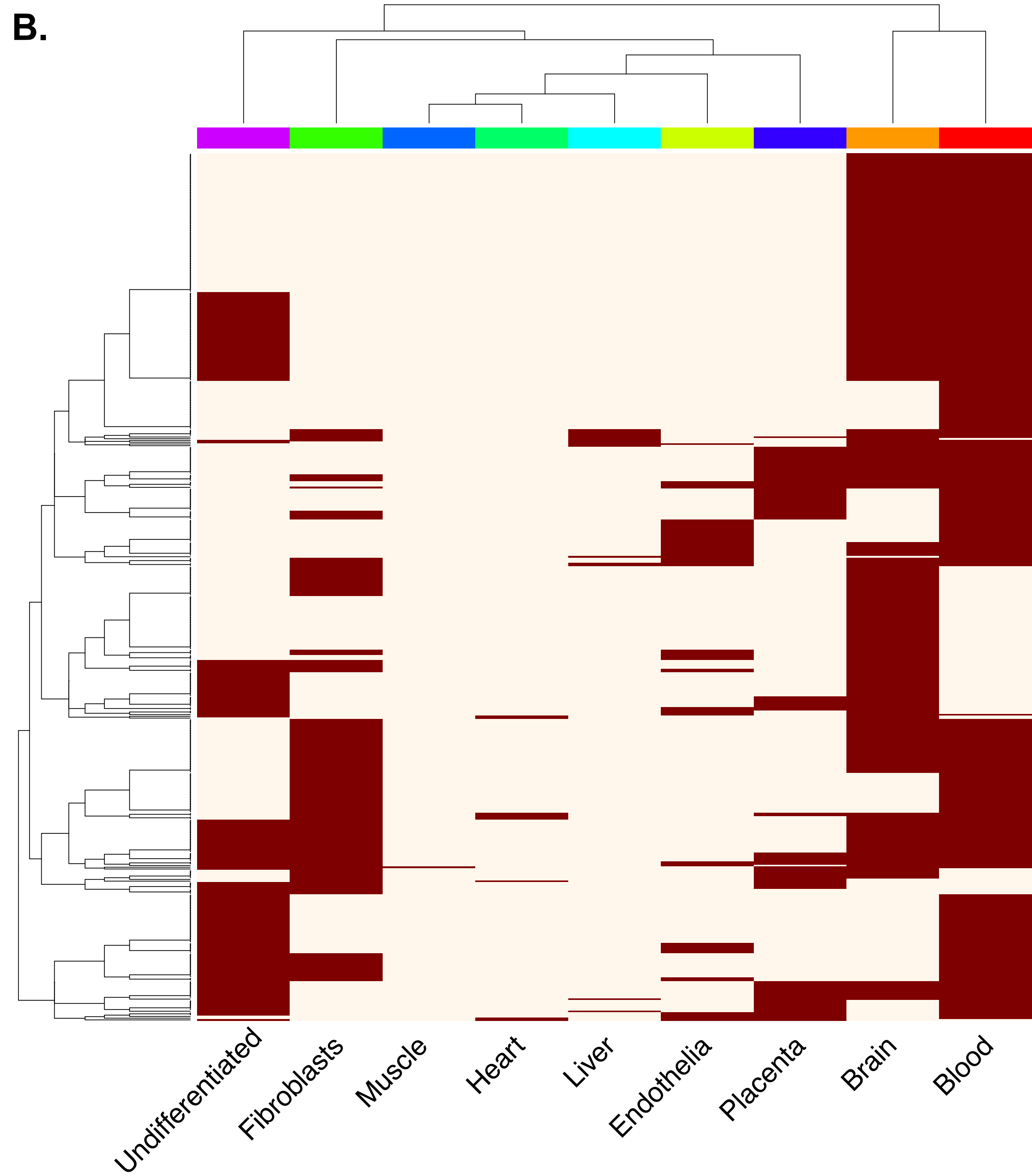

Supplement: S2 Fig — Each row is one of the 500 consistently DE genes and each column a tissue type (a) or macro-category (b). (PDF) [file pcbi.1009317.s002.pdf]

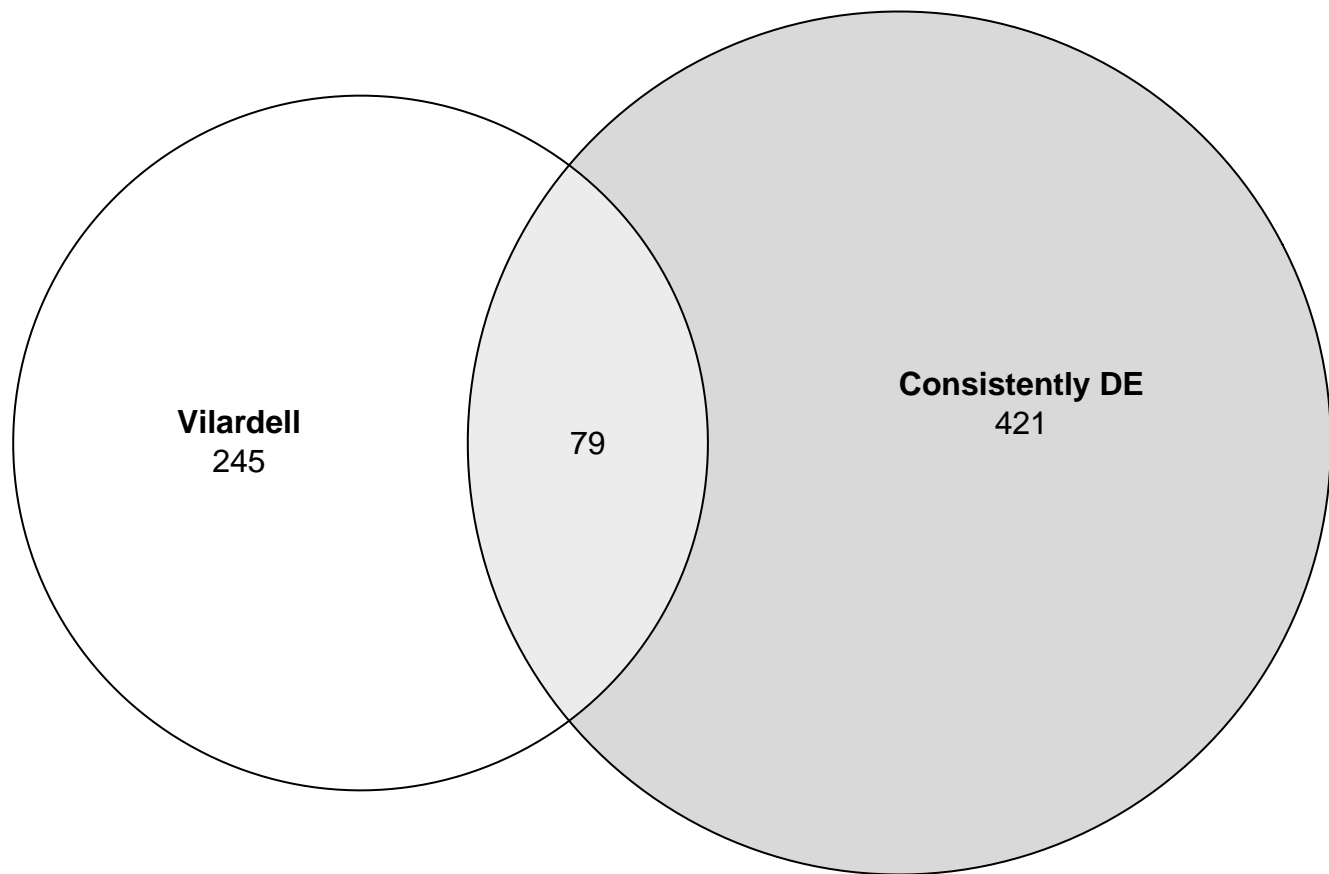

Supplement: S4 Fig — Venn Diagram showing the significant overlap of our consistently DE genes with the list of DS genes from Vilardell[7]. (PDF) [file pcbi.1009317.s004.pdf]

**b)**

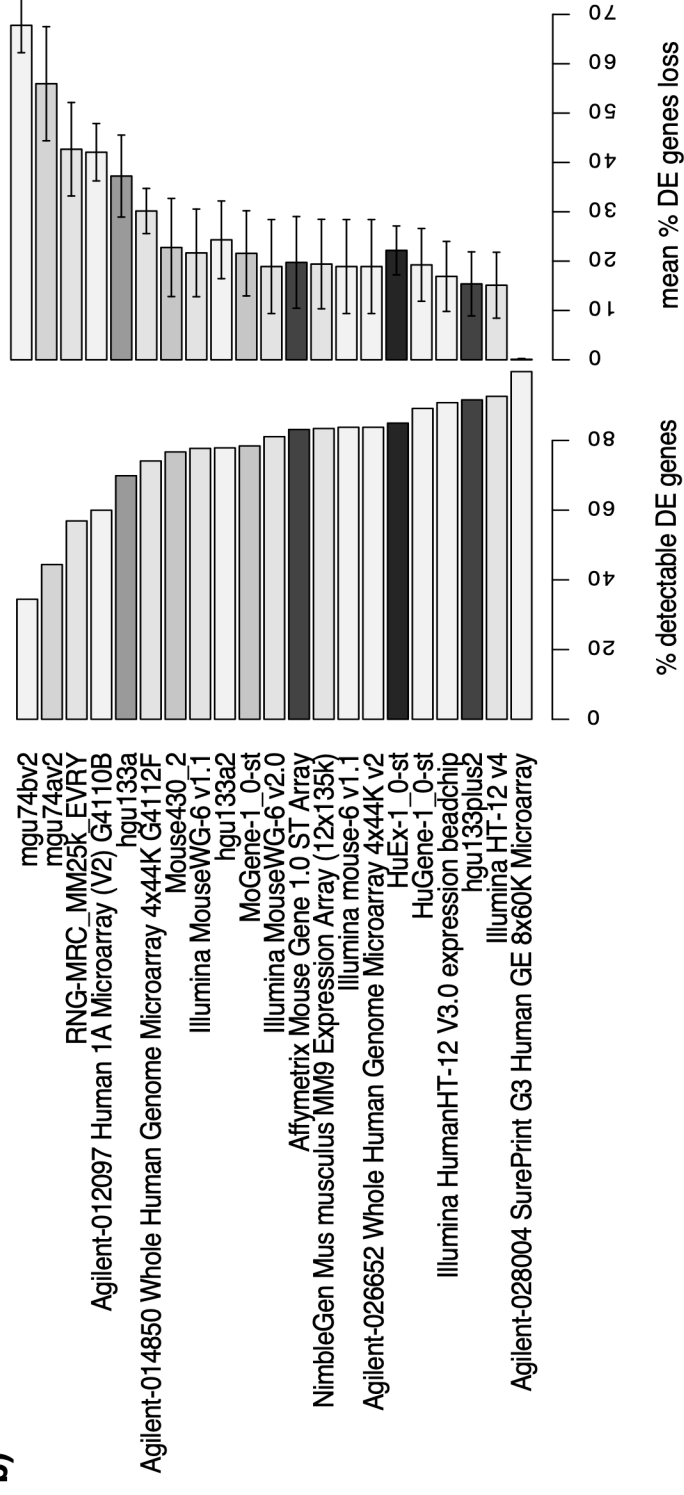

**a)**

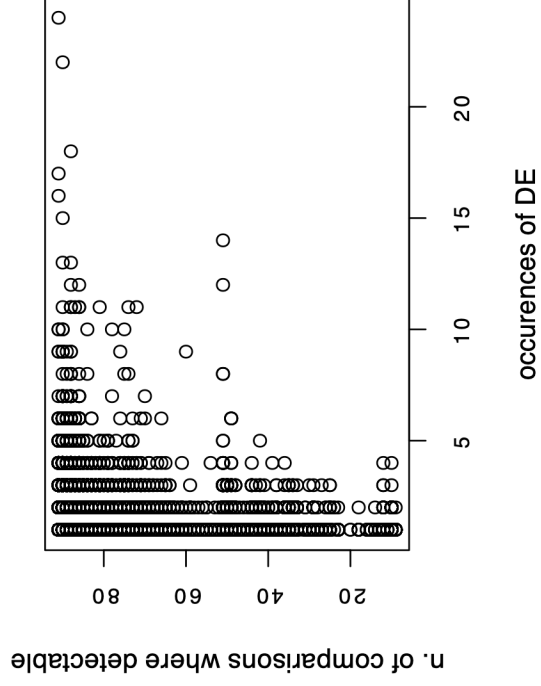

**c)**

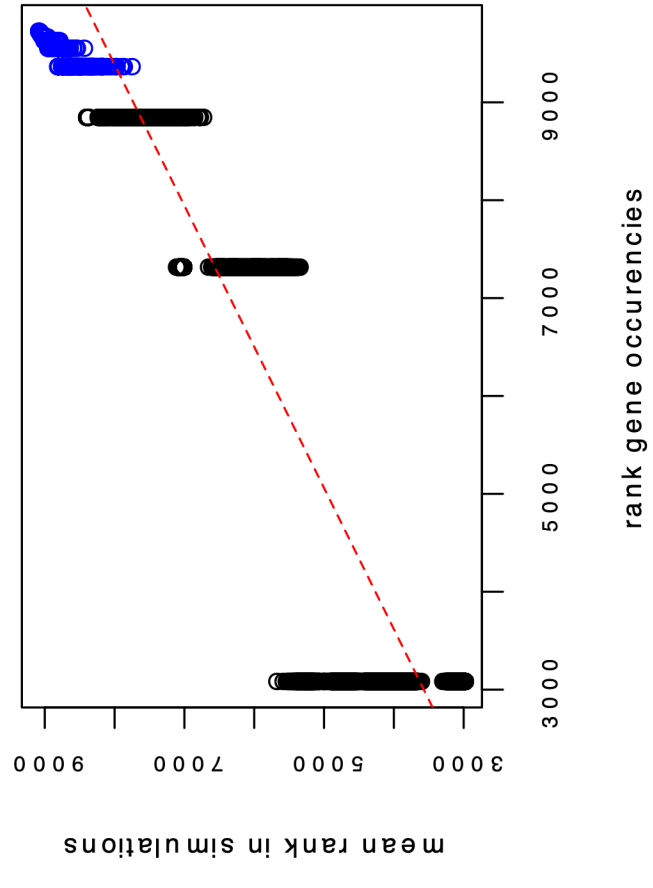

**d)**

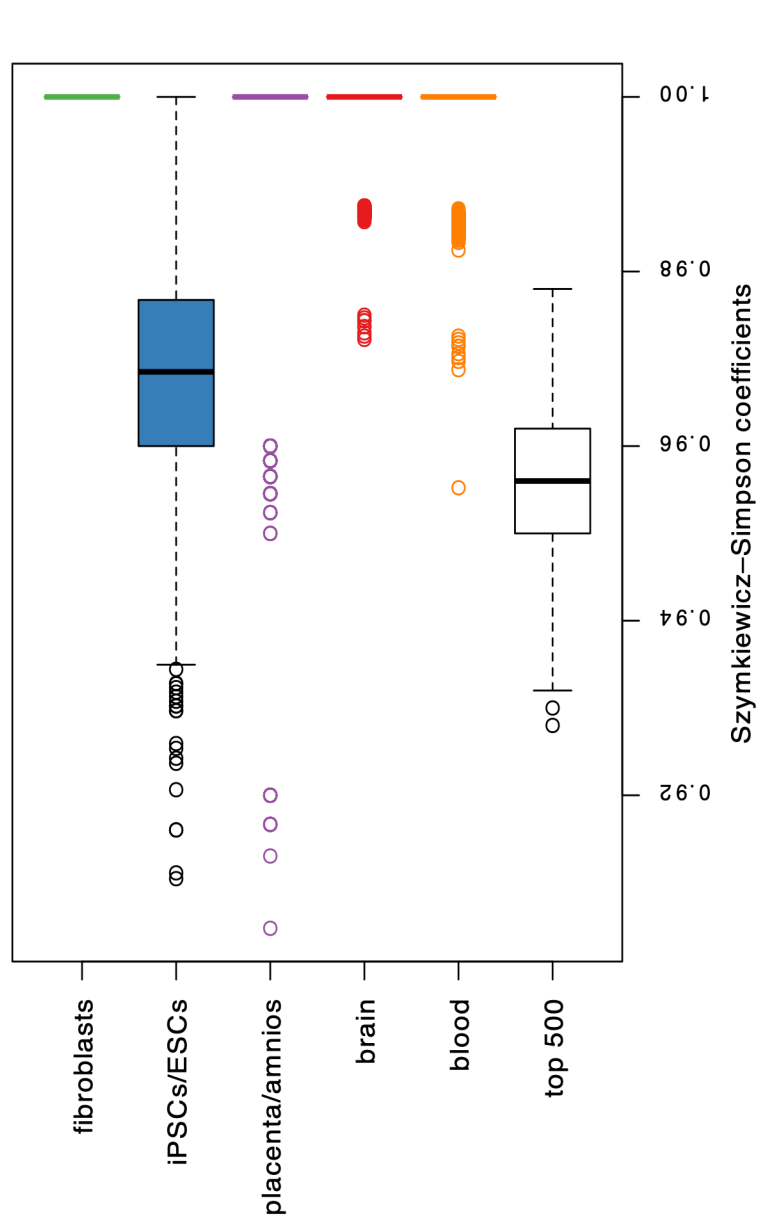

Supplement: S5 Fig — a) Plot showing the number a gene is found DE in our comparisons versus the number of times a gene is detectable. Each circle is one of the 9727 genes found DE in at least a comparison. b) Barplots showing the percentage of detectable DE genes in each platform (left) and the mean percentage of DE genes that would not be called “DE” due to coverage limitation in each platform. The error bars represent standard devation as this values were calculated from all the comparisons coming from RNA-seq experiments. The gradient of grays is proportional to the number of comparisons per each platform. c) Plot showing the positive correlation as indicated by the dashed red line between the actual rank of DE gene occurrences (x-axis) versus the mean rankings in the 1000 simulations. d) Boxplots showing the distributions of the Szymkiewicz-Simpson coefficients (overlap over the length of the smaller set) between the consistently DE genes and the preferentially DE genes in each macro-category with each of the respective simulated values from the 1000 permutations. (PDF) [file pcbi.1009317.s005.pdf]

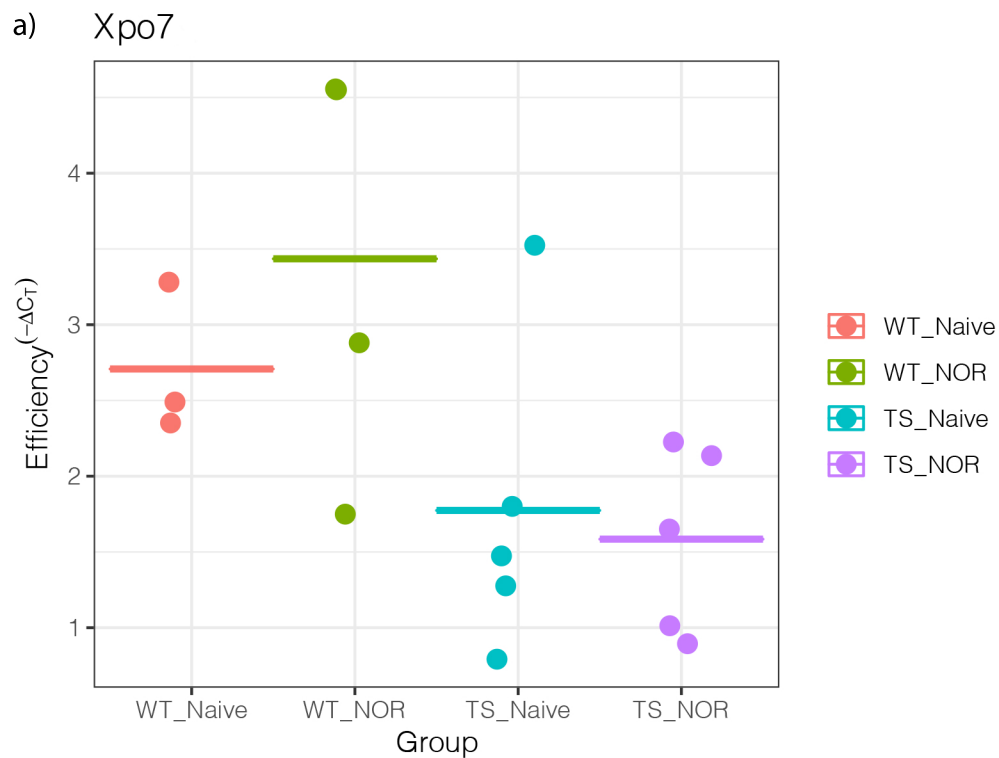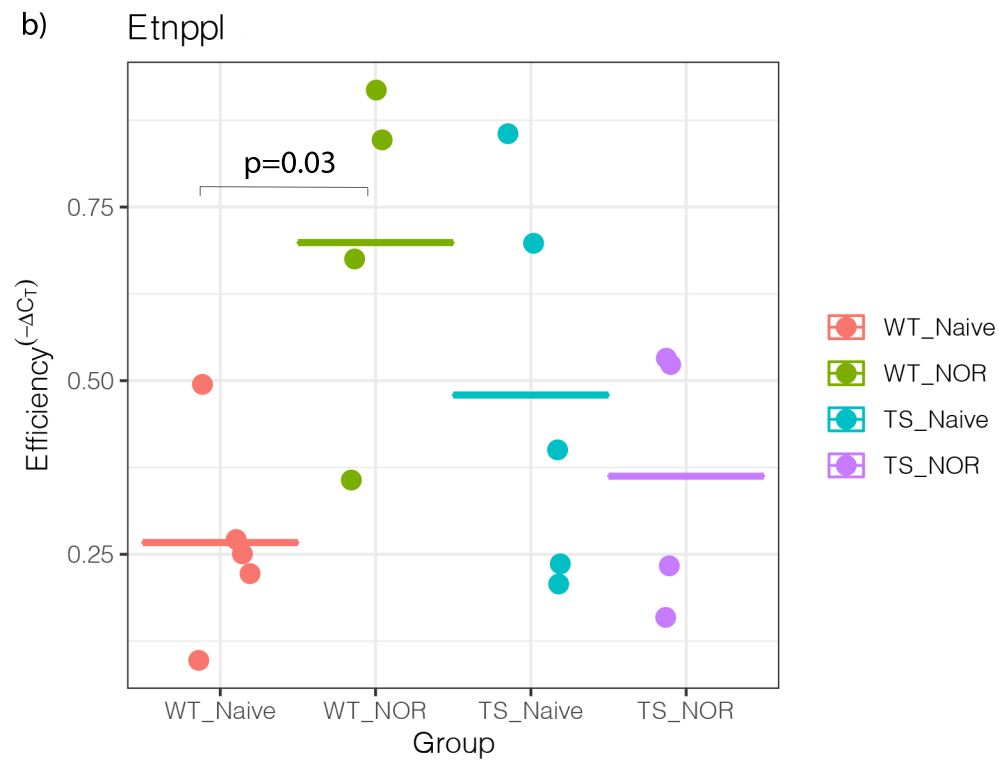

Supplement: S6 Fig — Quantitative PCR (qPCR) analysis of candidate genes on total RNA extracted from whole hippocampi (N = 4–5 per group) from mice sacrificed after conducting the Novel Object Recognition memory task (NOR) or not (Naive). Gene expression values are normalized to GAPDH. Each individual mouse is represented by a dot in the plot. Bars show the mean value of Efficiency^(-ΔCt) for each group. a) A decreasing tendency in Xpo7 expression levels in the Ts65Dn hippocampus was observed (p-value = 0.1111, Wilcoxon rank sum exact test) b) The NOR task triggers the expression of Etnppl in the WT but not in the Ts65Dn hippocampus (p-value = 0.03175, Wilcoxon rank sum exact test). (PDF) [file pcbi.1009317.s006.pdf]
