# Supplementary material for: Meta-analysis of transcriptomic data reveals clusters of consistently deregulated gene and disease ontologies in Down syndrome
Source: PLoS Comput Biol. 2021 Sep 27;17(9):e1009317. doi: 10.1371/journal.pcbi.1009317 (PMC8496798; doi:10.1371/journal.pcbi.1009317)
Supplement: S3 Fig — a) Tag cloud, where the size GEO/arrayExpress ID is proportional to the number of comparisons comparing trisomic versus disomic samples included in the study (e.g. different tissues, conditions, age, models, etc.). In red are the datasets that were included in the Vilardell et al. paper. b) As in A but with all the comparisons shown. (PDF) [file pcbi.1009317.s003.pdf]

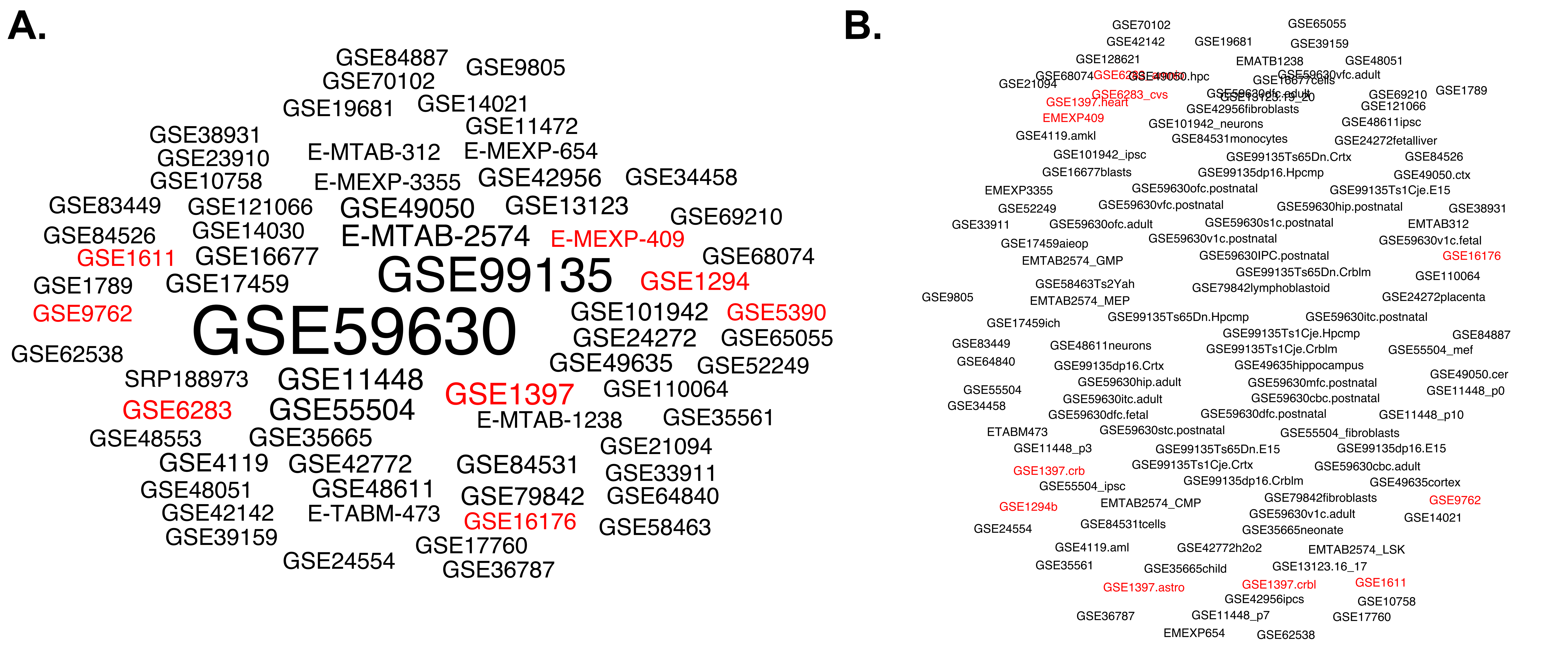

A.
GSE84887
GSE9805
GSE70102
GSE19681
GSE14021
GSE11472
GSE38931
GSE23910
GSE10758
E-MTAB-312
E-MEXP-654
E-MEXP-3355
GSE42956
GSE34458
GSE83449
GSE121066
GSE49050
GSE13123
GSE69210
GSE84526
GSE14030
E-MTAB-2574
E-MEXP-409
GSE1611
GSE16677
GSE68074
GSE1789
GSE17459
GSE99135
GSE1294
GSE9762
GSE59630
GSE101942
GSE5390
GSE24272
GSE65055
GSE62538
GSE49635
GSE52249
SRP188973
GSE11448
GSE1397
GSE110064
GSE6283
GSE55504
E-MTAB-1238
GSE35561
GSE48553
GSE35665
GSE21094
GSE4119
GSE42772
GSE84531
GSE33911
GSE48051
GSE48611
GSE79842
GSE64840
GSE42142
E-TABM-473
GSE16176
GSE58463
GSE39159
GSE17760
GSE24554
GSE36787
B.
GSE70102
GSE65055
GSE42142
GSE19681
GSE39159
GSE128621
EMATB1238
GSE48051
GSE21094
GSE68074
GSE59630vfc.adult
GSE6283_cvs
GSE69210
GSE1789
GSE1397.heart
GSE42956fibroblasts
GSE121066
EMEXP409
GSE101942_neurons
GSE48611ipsc
GSE4119.amkl
GSE84531monocytes
GSE24272fetalliver
GSE101942_ipsc
GSE99135Ts65Dn.Crtx
GSE84526
GSE16677blasts
GSE99135dp16.Hpcmp
GSE49050.ctx
EMEXP3355
GSE59630ofc.postnatal
GSE99135Ts1Cje.E15
GSE52249
GSE59630vfc.postnatal
GSE59630hip.postnatal
GSE38931
GSE33911
GSE59630ofc.adult
GSE59630s1c.postnatal
EMTAB312
GSE17459aieop
GSE59630v1c.postnatal
GSE59630v1c.fetal
EMTAB2574_GMP
GSE59630IPC.postnatal
GSE16176
GSE99135Ts65Dn.Crblm
GSE110064
GSE58463Ts2Yah
GSE79842lymphoblastoid
GSE9805
EMTAB2574_MEP
GSE24272placenta
GSE99135Ts65Dn.Hpcmp
GSE59630itc.postnatal
GSE17459ich
GSE99135Ts1Cje.Hpcmp
GSE84887
GSE83449
GSE48611neurons
GSE99135Ts1Cje.Crblm
GSE55504_mef
GSE64840
GSE99135dp16.Crtx
GSE49635hippocampus
GSE59630hip.adult
GSE59630mfc.postnatal
GSE49050.cer
GSE55504
GSE11448_p0
GSE59630itc.adult
GSE59630cbc.postnatal
GSE34458
GSE59630dfc.fetal
GSE59630dfc.postnatal
GSE11448_p10
ETABM473
GSE59630stc.postnatal
GSE55504_fibroblasts
GSE11448_p3
GSE99135Ts65Dn.E15
GSE99135dp16.E15
GSE1397.crb
GSE99135Ts1Cje.Crtx
GSE59630cbc.adult
GSE55504_ipsc
GSE99135dp16.Crblm
GSE49635cortex
GSE79842fibroblasts
GSE9762
GSE1294b
EMTAB2574_CMP
GSE59630v1c.adult
GSE14021
GSE24554
GSE84531tcells
GSE35665neonate
GSE4119.aml
GSE42772h2o2
EMTAB2574_LSK
GSE35561
GSE35665child
GSE13123.16_17
GSE1397.astro
GSE1397.crbl
GSE1611
GSE42956ipcs
GSE10758
GSE36787
GSE11448_p7
GSE17760
EMEXP654
GSE62538
