# Supplementary material for: Meta-analysis of transcriptomic data reveals clusters of consistently deregulated gene and disease ontologies in Down syndrome
Source: PLoS Comput Biol. 2021 Sep 27;17(9):e1009317. doi: 10.1371/journal.pcbi.1009317 (PMC8496798; doi:10.1371/journal.pcbi.1009317)
Supplement: S1 Table — (DOCX) [file pcbi.1009317.s011.docx]

**S1 Table.** Information on the dataset used in this meta-analysis.

| **Name** | **GEO/ArrayExpress** | **SRA_study** | **Species** | **Model** | **Platform** | **Cell_type** | **micro-category** | **macro-category** | **Design** | **PMID** |
| --- | --- | --- | --- | --- | --- | --- | --- | --- | --- | --- |
| GSE52249 | GSE52249 | SRP032928 | Homo sapiens | NA | Illumina HiSeq 2000 | iPSCs | iPSCs | Undifferentiated | 4 euploid; 3 trisomic | 24375627[[1]](https://www.zotero.org/google-docs/?uP0TQz) |
| GSE55504 | GSE55504 | SRP039348 | Homo sapiens | NA | Illumina HiSeq 2000 | Primary Fibroblasts | Fibroblasts | Fibroblasts | 4 euploid; 4 trisomic | 24740065[[2]](https://www.zotero.org/google-docs/?4g4unP) |
| GSE55504_fibroblasts | GSE55504 | SRP039348 | Homo sapiens | NA | Illumina HiSeq 2000 | Primary Fibroblasts | Fibroblasts | Fibroblasts | 8 euploid; 8 trisomic | 24740065 |
| GSE55504_ipsc | GSE55504 | SRP039348 | Homo sapiens | NA | Illumina HiSeq 2000 | iPSCs | iPSCs | Undifferentiated | 2 euploid; 1 trisomic | 24740065 |
| GSE55504_mef | GSE55504 | SRP039348 | Mus musculus | Ts65Dn | Illumina HiSeq 2000 | MEFs | Fibroblasts | Fibroblasts | 1 euploid; 1 trisomic | 24740065 |
| GSE42142 | GSE42142 | SRP017123 | Homo sapiens | NA | Illumina Genome Analyzer IIx | CVS | Placenta/amnios | Placenta/amnios | 4 euploid; 4 trisomic | 23754950[[3]](https://www.zotero.org/google-docs/?WZz745) |
| GSE64840 | GSE64840 | SRP051977 | Mus musculus | Ts65Dn | Illumina HiSeq 2500 | MEFs | Fibroblasts | Fibroblasts | 3 euploid; 3 trisomic | 26664707[[4]](https://www.zotero.org/google-docs/?lAOlrP) |
| GSE101942_ipsc | GSE101942 | SRP113668 | Homo sapiens | NA | Illumina HiSeq 2000 | iPSCs | iPSCs | Undifferentiated | 3 euploid; 3 trisomic | 29584757[[5]](https://www.zotero.org/google-docs/?qk3zth) |
| GSE101942_neurons | GSE101942 | SRP113668 | Homo sapiens | NA | Illumina HiSeq 2000 | iPSC-derived Neurons | Neurons | Neurons | 3 euploid; 3 trisomic | 29584757 |
| GSE6283_amnio | GSE6283 | NA | Homo sapiens | NA | hgu133plus2 | amniocytes | Placenta/amnios | Placenta/amnios | 3 euploid; 3 trisomic | 18253026[[6]](https://www.zotero.org/google-docs/?SWSoWi) |
| GSE6283_cvs | GSE6283 | NA | Homo sapiens | NA | hgu133plus2 | CVS | Placenta/amnios | Placenta/amnios | 6 euploid; 3 trisomic | 18253026 |
| GSE1294 | GSE1294 | NA | Mus musculus | Ts1Cje | mgu74av2 | Whole Brain | whole brain | Brain | 6 euploid; 6 trisomic | 15138197[[7]](https://www.zotero.org/google-docs/?WHwSGk) |
| GSE1294b | GSE1294 | NA | Mus musculus | Ts1Cje | mgu74bv2 | Whole Brain | whole brain | Brain | 6 euploid; 6 trisomic | 15138197 |
| GSE1611 | GSE1611 | NA | Mus musculus | Ts1Cje | mgu74av2 | Cerebellum | Cerebellum | Brain | 6 euploid; 6 trisomic | 15590701[[8]](https://www.zotero.org/google-docs/?GcuIx0) |
| GSE5390 | GSE5390 | NA | Homo sapiens | NA | hgu133a | Dorsolateral prefrontal cortex | Cortex | Brain | 8 euploid; 7 trisomic | 17950572[[9]](https://www.zotero.org/google-docs/?wh0m0P) |
| EMEXP409 | E-MEXP-409 | NA | Mus musculus | Tc1 | hgu133a | whole embryo | whole embryo | Brain | 1 euploid; 2 trisomic | 16179473[[10]](https://www.zotero.org/google-docs/?N6suaQ) |
| GSE1397.crb | GSE1397 | NA | Homo sapiens | NA | hgu133a | Cerebrum | Cerebrum | Brain | 4 euploid; 4 trisomic | 16420667[[11]](https://www.zotero.org/google-docs/?4mnVml) |
| GSE1397.crbl | GSE1397 | NA | Homo sapiens | NA | hgu133a | Cerebellum | Cerebellum | Brain | 3 euploid; 3 trisomic | 16420667 |
| GSE1397.astro | GSE1397 | NA | Homo sapiens | NA | hgu133a | astrocytes | astrocytes | Brain | 2 euploid; 2 trisomic | 16420667 |
| GSE1397.heart | GSE1397 | NA | Homo sapiens | NA | hgu133a | Heart | Heart | Heart | 2 euploid; 2 trisomic | 16420667 |
| GSE9762 | GSE9762 | NA | Homo sapiens | NA | hgu133plus2 | Primary Fibroblasts | Fibroblasts | Fibroblasts | 5 euploid; 5 trisomic | NA |
| GSE16176 | GSE16176 | NA | Homo sapiens | NA | hgu133plus2 | amniotic fluid | Placenta/amnios | Placenta/amnios | 7 euploid; 7 trisomic | 19474297[[12]](https://www.zotero.org/google-docs/?DKhSph) |
| EMEXP654 | E-MEXP-654 | NA | Mus musculus | transchromosomic cells 47-1 | mgu74av2 | mESCs | ESCs | Undifferentiated | 4 euploid; 4 trisomic | 20569505[[13]](https://www.zotero.org/google-docs/?7SzaMf) |
| GSE35561 | GSE35561 | NA | Homo sapiens | NA | HuGene-1_0-st | iPSCs | iPSCs | Blood/bone marrow | 3 euploid; 3 trisomic | 23045704[[14]](https://www.zotero.org/google-docs/?7qBxaz) |
| GSE39159 | GSE39159 | NA | Mus musculus | Ts65Dn | Affymetrix Mouse Gene 1.0 ST Array | Soleus muscles | Muscle | Muscle | 4 euploid; 4 trisomic | 23115123[[15]](https://www.zotero.org/google-docs/?TT2dXG) |
| GSE110064 | GSE110064 | NA | Homo sapiens | NA | hgu133plus2 | B-cells | B-cells | Blood/bone marrow | 3 euploid; 3 trisomic | 29789608[[16]](https://www.zotero.org/google-docs/?kSMlUU) |
| GSE99135dp16.E15 | GSE99135 | NA | Mus musculus | Dp16 | Affymetrix Mouse Gene 1.0 ST Array | dp16.E15 | Forebrain | Brain | 6 euploid; 6 trisomic | 29716957[[17]](https://www.zotero.org/google-docs/?5HJMv2) |
| GSE99135Ts65Dn.E15 | GSE99135 | NA | Mus musculus | Ts65Dn | Affymetrix Mouse Gene 1.0 ST Array | Ts65Dn.E15 | Forebrain | Brain | 7 euploid; 6 trisomic | 29716957 |
| GSE99135Ts1Cje.E15 | GSE99135 | NA | Mus musculus | Ts1Cje | Affymetrix Mouse Gene 1.0 ST Array | Ts1Cje.E15 | Forebrain | Brain | 5 euploid; 5 trisomic | 29716957 |
| GSE99135dp16.Crblm | GSE99135 | NA | Mus musculus | Dp16 | Affymetrix Mouse Gene 1.0 ST Array | dp16.crblm | Cerebellum | Brain | 5 euploid; 5 trisomic | 29716957 |
| GSE99135Ts65Dn.Crblm | GSE99135 | NA | Mus musculus | Ts65Dn | Affymetrix Mouse Gene 1.0 ST Array | Ts65Dn.crblm | Cerebellum | Brain | 5 euploid; 5 trisomic | 29716957 |
| GSE99135Ts1Cje.Crblm | GSE99135 | NA | Mus musculus | Ts1Cje | Affymetrix Mouse Gene 1.0 ST Array | Ts1Cje.crblm | Cerebellum | Brain | 5 euploid; 5 trisomic | 29716957 |
| GSE99135dp16.Crtx | GSE99135 | NA | Mus musculus | Dp16 | Affymetrix Mouse Gene 1.0 ST Array | dp16.crtx | Cortex | Brain | 5 euploid; 5 trisomic | 29716957 |
| GSE99135Ts65Dn.Crtx | GSE99135 | NA | Mus musculus | Ts65Dn | Affymetrix Mouse Gene 1.0 ST Array | Ts65Dn.crtx | Cortex | Brain | 5 euploid; 5 trisomic | 29716957 |
| GSE99135Ts1Cje.Crtx | GSE99135 | NA | Mus musculus | Ts1Cje | Affymetrix Mouse Gene 1.0 ST Array | Ts1Cje.crtx | Cortex | Brain | 5 euploid; 6 trisomic | 29716957 |
| GSE99135dp16.Hpcmp | GSE99135 | NA | Mus musculus | Dp16 | Affymetrix Mouse Gene 1.0 ST Array | dp16.Hpcmp | Hippocampus | Brain | 5 euploid; 5 trisomic | 29716957 |
| GSE99135Ts65Dn.Hpcmp | GSE99135 | NA | Mus musculus | Ts65Dn | Affymetrix Mouse Gene 1.0 ST Array | Ts65Dn.Hpcmp | Hippocampus | Brain | 5 euploid; 5 trisomic | 29716957 |
| GSE99135Ts1Cje.Hpcmp | GSE99135 | NA | Mus musculus | Ts1Cje | Affymetrix Mouse Gene 1.0 ST Array | Ts1Cje.Hpcmp | Hippocampus | Brain | 7 euploid; 5 trisomic | 29716957 |
| GSE69210 | GSE69210 | NA | Homo sapiens | NA | Agilent-026652 Whole Human Genome Microarray 4x44K v2 | Thymus | Thymus | Blood/bone marrow | 10 euploid; 10 trisomic | 26848775[[18]](https://www.zotero.org/google-docs/?NnZiTk) |
| GSE84887 | GSE84887 | NA | Homo sapiens | NA | hgu133plus2 | NPCs | NPCs | Brain | 3 euploid; 5 trisomic | 27618722[[19]](https://www.zotero.org/google-docs/?hqOXVp) |
| GSE83449 | GSE83449 | NA | Homo sapiens | NA | hgu133a | AMKL | Blood/bone marrow | Blood/bone marrow | 31 euploid; 22 trisomic | 16492768[[20]](https://www.zotero.org/google-docs/?EBgicE) |
| GSE59630dfc.fetal | GSE59630 | NA | Homo sapiens | NA | HuEx-1_0-st | DFC | Cortex | Brain | 2 euploid; 2 trisomic | 26924435[[21]](https://www.zotero.org/google-docs/?dDkAwk) |
| GSE59630dfc.postnatal | GSE59630 | NA | Homo sapiens | NA | HuEx-1_0-st | DFC | Cortex | Brain | 7 euploid; 7 trisomic | 26924435 |
| GSE59630dfc.adult | GSE59630 | NA | Homo sapiens | NA | HuEx-1_0-st | DFC | Cortex | Brain | 3 euploid; 3 trisomic | 26924435 |
| GSE59630cbc.postnatal | GSE59630 | NA | Homo sapiens | NA | HuEx-1_0-st | CBC | Cerebellum | Brain | 8 euploid; 8 trisomic | 26924435 |
| GSE59630cbc.adult | GSE59630 | NA | Homo sapiens | NA | HuEx-1_0-st | CBC | Cerebellum | Brain | 2 euploid; 2 trisomic | 26924435 |
| GSE59630hip.postnatal | GSE59630 | NA | Homo sapiens | NA | HuEx-1_0-st | HIP | Hippocampus | Brain | 2 euploid; 2 trisomic | 26924435 |
| GSE59630hip.adult | GSE59630 | NA | Homo sapiens | NA | HuEx-1_0-st | HIP | Hippocampus | Brain | 1 euploid; 1 trisomic | 26924435 |
| GSE59630v1c.fetal | GSE59630 | NA | Homo sapiens | NA | HuEx-1_0-st | V1C | Cortex | Brain | 2 euploid; 2 trisomic | 26924435 |
| GSE59630v1c.postnatal | GSE59630 | NA | Homo sapiens | NA | HuEx-1_0-st | V1C | Cortex | Brain | 7 euploid; 7 trisomic | 26924435 |
| GSE59630v1c.adult | GSE59630 | NA | Homo sapiens | NA | HuEx-1_0-st | V1C | Cortex | Brain | 2 euploid; 2 trisomic | 26924435 |
| GSE59630itc.postnatal | GSE59630 | NA | Homo sapiens | NA | HuEx-1_0-st | ITC | Cortex | Brain | 3 euploid; 3 trisomic | 26924435 |
| GSE59630itc.adult | GSE59630 | NA | Homo sapiens | NA | HuEx-1_0-st | ITC | Cortex | Brain | 2 euploid; 2 trisomic | 26924435 |
| GSE59630stc.postnatal | GSE59630 | NA | Homo sapiens | NA | HuEx-1_0-st | STC | Cortex | Brain | 1 euploid; 1 trisomic | 26924435 |
| GSE59630IPC.postnatal | GSE59630 | NA | Homo sapiens | NA | HuEx-1_0-st | IPC | Cortex | Brain | 1 euploid; 1 trisomic | 26924435 |
| GSE59630s1c.postnatal | GSE59630 | NA | Homo sapiens | NA | HuEx-1_0-st | S1C | Cortex | Brain | 1 euploid; 1 trisomic | 26924435 |
| GSE59630mfc.postnatal | GSE59630 | NA | Homo sapiens | NA | HuEx-1_0-st | MFC | Cortex | Brain | 1 euploid; 1 trisomic | 26924435 |
| GSE59630vfc.postnatal | GSE59630 | NA | Homo sapiens | NA | HuEx-1_0-st | VFC | Cortex | Brain | 5 euploid; 5 trisomic | 26924435 |
| GSE59630vfc.adult | GSE59630 | NA | Homo sapiens | NA | HuEx-1_0-st | VFC | Cortex | Brain | 2 euploid; 2 trisomic | 26924435 |
| GSE59630ofc.postnatal | GSE59630 | NA | Homo sapiens | NA | HuEx-1_0-st | OFC | Cortex | Brain | 4 euploid; 4 trisomic | 26924435 |
| GSE59630ofc.adult | GSE59630 | NA | Homo sapiens | NA | HuEx-1_0-st | OFC | Cortex | Brain | 2 euploid; 2 trisomic | 26924435 |
| GSE58463Ts2Yah | GSE58463 | NA | Mus musculus | Ts2Yah | Illumina MouseWG-6 v2.0 | gastrocnemius muscles | Muscle | Muscle | 5 euploid; 6 trisomic | NA |
| GSE65055 | GSE65055 | NA | Homo sapiens | NA | HuGene-2_0-st | CVS | Placenta/amnios | Placenta/amnios | 5 euploid; 5 trisomic | NA |
| GSE70102 | GSE70102 | NA | Homo sapiens | NA | hgu133plus2 | maternal fetal interface | Placenta/amnios | Placenta/amnios | 4 euploid; 8 trisomic | 27328057[[22]](https://www.zotero.org/google-docs/?lk4FF8) |
| GSE68074 | GSE68074 | NA | Mus musculus | Ts65Dn | Agilent-028005 SurePrint G3 Mouse GE 8x60K Microarray | Hippocampus | Hippocampus | Brain | 4 euploid; 3 trisomic | 26546125[[23]](https://www.zotero.org/google-docs/?kPozwj) |
| GSE36787 | GSE36787 | NA | Homo sapiens | NA | HuGene-1_0-st | CD43+/41+/235+ progenitors | iPSCs | Blood/bone marrow | 2 euploid; 6 trisomic | 25621499[[24]](https://www.zotero.org/google-docs/?wbj7F1) |
| GSE62538 | GSE62538 | NA | Mus musculus | Ts1Cje | MoGene-1_0-st | Ts1Cje.E15 | Forebrain | Brain | 5 euploid; 5 trisomic | 25975229[[25]](https://www.zotero.org/google-docs/?5EoFIc) |
| GSE49050.cer | GSE49050 | NA | Mus musculus | Ts1Cje | Mouse430_2 | Ts1Cje.crblm | Cerebellum | Brain | 5 euploid; 5 trisomic | 25052193[[26]](https://www.zotero.org/google-docs/?Abq48j) |
| GSE49050.ctx | GSE49050 | NA | Mus musculus | Ts1Cje | Mouse430_2 | Ts1Cje.crtx | Cortex | Brain | 5 euploid; 5 trisomic | 25052193 |
| GSE49050.hpc | GSE49050 | NA | Mus musculus | Ts1Cje | Mouse430_2 | Ts1Cje.Hpcmp | Hippocampus | Brain | 5 euploid; 5 trisomic | 25052193 |
| GSE49635cortex | GSE49635 | NA | Mus musculus | Ts1Cje | MoGene-1_0-st | Ts1Cje.crtx | Cortex | Brain | 5 euploid; 6 trisomic | 24916381[[27]](https://www.zotero.org/google-docs/?QHMNUU) |
| GSE49635hippocampus | GSE49635 | NA | Mus musculus | Ts1Cje | MoGene-1_0-st | Ts1Cje.Hpcmp | Hippocampus | Brain | 5 euploid; 6 trisomic | 24916381 |
| GSE48611ipsc | GSE48611 | NA | Homo sapiens | NA | hgu133plus2 | iPSCs | iPSCs | Undifferentiated | 3 euploid; 6 trisomic | 23716668[[28]](https://www.zotero.org/google-docs/?PQCyFt) |
| GSE48611neurons | GSE48611 | NA | Homo sapiens | NA | hgu133plus2 | Neurons | Neurons | Brain | 3 euploid; 6 trisomic | 23716668 |
| GSE48051 | GSE48051 | NA | Homo sapiens | NA | Agilent-014850 Whole Human Genome Microarray 4x44K G4112F | amniocyte | Placenta/amnios | Placenta/amnios | 9 euploid; 10 trisomic | 24066117[[29]](https://www.zotero.org/google-docs/?BZvSbJ) |
| GSE42956ipcs | GSE42956 | NA | Homo sapiens | NA | Illumina HT-12 v4 | iPSCs | iPSCs | Undifferentiated | 15 euploid; 12 trisomic | 23225669[[30]](https://www.zotero.org/google-docs/?TIrNij) |
| GSE42956fibroblasts | GSE42956 | NA | Homo sapiens | NA | Illumina HT-12 v4 | fibroblasts | Fibroblasts | Fibroblasts | 15 euploid; 6 trisomic | 23225669 |
| GSE42772 | GSE42772 | NA | Homo sapiens | NA | Illumina humanRef-8 v2.0 expression beadchip | astrocytes | astrocytes | Cultures | 3 euploid; 5 trisomic | 23312288[[31]](https://www.zotero.org/google-docs/?zVgJLM) |
| GSE42772h2o2 | GSE42772 | NA | Homo sapiens | NA | Illumina humanRef-8 v2.0 expression beadchip | astrocytes | astrocytes | Cultures | 3 euploid; 5 trisomic | 23312288 |
| GSE38931 | GSE38931 | NA | Homo sapiens | NA | Illumina HumanHT-12 V4.0 expression beadchip | iPSCs | iPSCs | Undifferentiated | 4 euploid; 2 trisomic | 23084023[[32]](https://www.zotero.org/google-docs/?saZFrp) |
| GSE34458 | GSE34458 | NA | Homo sapiens | NA | Illumina HumanHT-12 V3.0 expression beadchip | lymphoblastoid cell line | Blood/bone marrow | Blood/bone marrow | 12 euploid; 11 trisomic | 22912673[[33]](https://www.zotero.org/google-docs/?XqmFDt) |
| GSE35665child | GSE35665 | NA | Homo sapiens | NA | HuEx-1_0-st | PBMC | Blood/bone marrow | Blood/bone marrow | 15 euploid; 10 trisomic | 23155455[[34]](https://www.zotero.org/google-docs/?WP4B9T) |
| GSE35665neonate | GSE35665 | NA | Homo sapiens | NA | HuEx-1_0-st | PBMC | Blood/bone marrow | Blood/bone marrow | 7 euploid; 5 trisomic | 23155455 |
| GSE33911 | GSE33911 | NA | Homo sapiens | NA | Agilent-028004 SurePrint G3 Human GE 8x60K Microarray | iPSCs | iPSCs | Undifferentiated | 3 euploid; 3 trisomic | NA |
| GSE23910 | GSE23910 | NA | Homo sapiens | NA | Agilent-014850 Whole Human Genome Microarray 4x44K G4112F | thymus | Thymus | Blood/bone marrow | 4 euploid; 4 trisomic | 21856934[[35]](https://www.zotero.org/google-docs/?fkpCg8) |
| GSE21094 | GSE21094 | NA | Homo sapiens | NA | hgu133plus2 | BM | Blood/bone marrow | Blood/bone marrow | 26 euploid; 26 trisomic | 21647151[[36]](https://www.zotero.org/google-docs/?qcW8yO) |
| GSE24272fetalliver | GSE24272 | NA | Mus musculus | Ts1Cje | NimbleGen Mus musculus MM9 Expression Array (12x135k) | fetal liver | Fetal liver | Liver | 12 euploid; 13 trisomic | 21533146[[37]](https://www.zotero.org/google-docs/?GP1SrR) |
| GSE24272placenta | GSE24272 | NA | Mus musculus | Ts1Cje | NimbleGen Mus musculus MM9 Expression Array (12x135k) | placenta | Placenta/amnios | Placenta/amnios | 12 euploid; 13 trisomic | 21533146 |
| GSE24554 | GSE24554 | NA | Mus musculus | Ts65Dn | Illumina MouseWG-6 v2.0 | Mandible precursor | Mandible precursor | Undifferentiated | 11 euploid; 13 trisomic | NA |
| GSE16677blasts | GSE16677 | NA | Homo sapiens | NA | hgu133plus2 | AMKL sorted blast | Blood/bone marrow | Blood/bone marrow | 5 euploid; 6 trisomic | 20679399[[38]](https://www.zotero.org/google-docs/?YiKUee) |
| GSE16677cells | GSE16677 | NA | Homo sapiens | NA | hgu133plus2 | AMKL cell line | Blood/bone marrow | Blood/bone marrow | 4 euploid; 2 trisomic | 20679399 |
| GSE17760 | GSE17760 | NA | Mus musculus | Ts1Cje | Mouse430_2 | neurospheres | NPCs | Brain | 3 euploid; 3 trisomic | 20661276[[39]](https://www.zotero.org/google-docs/?838RsS) |
| GSE17459aieop | GSE17459 | NA | Homo sapiens | NA | hgu133plus2 | white blood cells | Blood/bone marrow | Blood/bone marrow | 72 euploid; 25 trisomic | 19965641[[40]](https://www.zotero.org/google-docs/?TIqcZR) |
| GSE17459ich | GSE17459 | NA | Homo sapiens | NA | hgu133plus2 | white blood cells | Blood/bone marrow | Blood/bone marrow | 9 euploid; 6 trisomic | 19965641 |
| GSE19681 | GSE19681 | NA | Homo sapiens | NA | hgu133plus2 | primary leukemic blasts | Blood/bone marrow | Blood/bone marrow | 2 euploid; 3 trisomic | 20194440[[41]](https://www.zotero.org/google-docs/?erBOT5) |
| GSE9805 | GSE9805 | NA | Mus musculus | Ts1Cje | RNG-MRC_MM25k_EVRY | NPCs | Cortex | Brain | 3 euploid; 3 trisomic | 19472221[[42]](https://www.zotero.org/google-docs/?WFuw0b) |
| GSE11448_p0 | GSE11448 | NA | Mus musculus | Ts1Cje | RNG-MRC_MM25k_EVRY | P0.Cerebellum | Cerebellum | Brain | 6 euploid; 6 trisomic | 19331679[[43]](https://www.zotero.org/google-docs/?PSJAie) |
| GSE11448_p3 | GSE11448 | NA | Mus musculus | Ts1Cje | RNG-MRC_MM25k_EVRY | P3.Cerebellum | Cerebellum | Brain | 9 euploid; 8 trisomic | 19331679 |
| GSE11448_p7 | GSE11448 | NA | Mus musculus | Ts1Cje | RNG-MRC_MM25k_EVRY | P7.Cerebellum | Cerebellum | Brain | 7 euploid; 8 trisomic | 19331679 |
| GSE11448_p10 | GSE11448 | NA | Mus musculus | Ts1Cje | RNG-MRC_MM25k_EVRY | P10.Cerebellum | Cerebellum | Brain | 6 euploid; 6 trisomic | 19331679 |
| GSE14021 | GSE14021 | NA | Mus musculus | TgDyrk1A | Agilent-012694 Whole Mouse Genome G4122A | E12.5.telencephalon | Cortex | Brain | 2 euploid; 2 trisomic | 19218269[[44]](https://www.zotero.org/google-docs/?BwKuQK) |
| GSE14030 | GSE14030 | NA | Mus musculus | TgDyrk1A-N18 | Agilent-012694 Whole Mouse Genome G4122A | neuroblastoma cell line | Neuroblastoma | Brain | 5 euploid; 5 trisomic | 20457675[[45]](https://www.zotero.org/google-docs/?InVbMd) |
| GSE13123.16_17 | GSE13123 | NA | Mus musculus | Ts1Cje/Ts65Dn | Illumina MouseWG-6 v1.1 | 16-17.dpp.sperm cells | Sperm Cells | Sperm Cells | 3 euploid; 3+3 trisomic | 19639331[[46]](https://www.zotero.org/google-docs/?z0f1fs) |
| GSE13123.19_20 | GSE13123 | NA | Mus musculus | Ts1Cje/Ts65Dn | Illumina MouseWG-6 v1.1 | 19-20.dpp.sperm cells | Sperm Cells | Sperm Cells | 3 euploid; 3+3 trisomic | 19639331 |
| GSE11472 | GSE11472 | NA | Mus musculus | Ts1Cje | Illumina mouse-6 v1.1 | cerebellar external granular layer | Cerebellum | Brain | 9 euploid; 9 trisomic | 19331679[[43]](https://www.zotero.org/google-docs/?mRfg8M) |
| GSE10758 | GSE10758 | NA | Homo sapiens | NA | Agilent-012097 Human 1A Microarray (V2) G4110B | amniocytes | Placenta/amnios | Placenta/amnios | 6 euploid; 4 trisomic | 18594911[[47]](https://www.zotero.org/google-docs/?rqWYH3) |
| GSE1789 | GSE1789 | NA | Homo sapiens | NA | hgu133a | fetal heart explant | Heart | Heart | 3 euploid; 5 trisomic | 17683628[[48]](https://www.zotero.org/google-docs/?vEemog) |
| GSE4119.amkl | GSE4119 | NA | Homo sapiens | NA | hgu133a | AMKL mononuclear cells | Blood/bone marrow | Blood/bone marrow | 38 euploid; 23 trisomic | 16492768[[20]](https://www.zotero.org/google-docs/?ZuMkiF) |
| GSE4119.aml | GSE4119 | NA | Homo sapiens | NA | hgu133a | AML mononuclear cells | Blood/bone marrow | Blood/bone marrow | 9 euploid; 2 trisomic | 16492768 |
| ETABM473 | E-TABM-473 | NA | Mus musculus | Tc1 | Illumina MouseWG-6 v1.1 | hepatocytes | Liver | Liver | 4 euploid; 4 trisomic | 18787134[[49]](https://www.zotero.org/google-docs/?ttMcWy) |
| EMTAB312 | E-MTAB-312 | NA | Homo sapiens | NA | hgu133a2 | EPCs | Endothelia | Endothelia | 4 euploid; 2 trisomic | 20836844[[50]](https://www.zotero.org/google-docs/?PHVfQA) |
| EMEXP3355 | E-MEXP-3355 | NA | Mus musculus | Ts65Dn | MoGene-1_0-st | Heart | Heart | Heart | 5 euploid; 5 trisomic | 22693452[[51]](https://www.zotero.org/google-docs/?WAEkZT) |
| EMATB1238 | E-MTAB-1238 | NA | Homo sapiens | NA | hgu133plus2 | LCLs | Blood/bone marrow | Blood/bone marrow | 6 euploid; 6 trisomic | 23830204[[52]](https://www.zotero.org/google-docs/?Ro2KsO) |
| EMTAB2574_GMP | E-MTAB-2574 | NA | Mus musculus | Ts65Dn | Illumina MouseWG-6 v2.0 | GMP | Blood/bone marrow | Blood/bone marrow | 3 euploid; 3 trisomic | 25973911[[53]](https://www.zotero.org/google-docs/?FoUZJP) |
| EMTAB2574_MEP | E-MTAB-2574 | NA | Mus musculus | Ts65Dn | Illumina MouseWG-6 v2.0 | MEP | Blood/bone marrow | Blood/bone marrow | 3 euploid; 3 trisomic | 25973911 |
| EMTAB2574_LSK | E-MTAB-2574 | NA | Mus musculus | Ts65Dn | Illumina MouseWG-6 v2.0 | LSK | Blood/bone marrow | Blood/bone marrow | 3 euploid; 3 trisomic | 25973911 |
| EMTAB2574_CMP | E-MTAB-2574 | NA | Mus musculus | Ts65Dn | Illumina MouseWG-6 v2.0 | CMP | Blood/bone marrow | Blood/bone marrow | 3 euploid; 3 trisomic | 25973911 |
| GSE48553 | GSE48553 | SRP026566 | Mus musculus | Ts1Rhr | Illumina HiSeq 2000 | proB-cells cells at passage 1 | Blood/bone marrow | Blood/bone marrow | 3 euploid; 3 trisomic | 24747640[[54]](https://www.zotero.org/google-docs/?ho8S6R) |
| GSE79842fibroblasts | GSE79842 | SRP072769 | Homo sapiens | NA | Illumina HiSeq 2500 | fibroblasts | Fibroblasts | Fibroblasts | 6 euploid; 6 trisomic | 27472900[[55]](https://www.zotero.org/google-docs/?xZyShW) |
| GSE79842lymphoblastoid | GSE79842 | SRP072769 | Homo sapiens | NA | Illumina HiSeq 2500 | lymphoblastoid | Blood/bone marrow | Blood/bone marrow | 3 euploid; 3 trisomic | 27472900 |
| GSE84526 | GSE84526 | SRP078911 | Mus musculus | Dp16 | Illumina HiSeq 2500 | lineage negative, Sca1 positive, c-kit positive (LSK) cells | Blood/bone marrow | Blood/bone marrow | 3 euploid; 3 trisomic | 27472900 |
| GSE84531monocytes | GSE84531 | SRP078912 | Homo sapiens | NA | Illumina HiSeq 4000 | Monocytes | Blood/bone marrow | Blood/bone marrow | 7 euploid; 10 trisomic | 27472900 |
| GSE84531tcells | GSE84531 | SRP078912 | Homo sapiens | NA | Illumina HiSeq 4000 | T cells | Blood/bone marrow | Blood/bone marrow | 7 euploid; 10 trisomic | 27472900 |
| GSE121066 | GSE121066 | SRP164925 | Mus musculus | Ts1Rhr | Illumina NextSeq 500 | primary mouse proB_Ts1Rhr cells expanded in culture | Blood/bone marrow | Blood/bone marrow | 3 euploid; 3 trisomic | 30428356[[56]](https://www.zotero.org/google-docs/?409CHO) |
| GSE128621 | SRP188973 | SRP188973 | Homo sapiens | NA | Illumina HiSeq 4000 | white blood cells | Blood/bone marrow | Blood/bone marrow | 6+3 euploid; 8+2 trisomic | 31628327[[57]](https://www.zotero.org/google-docs/?duaPJZ) |

References for S1 Table

[1. Hibaoui Y, Grad I, Letourneau A, Sailani MR, Dahoun S, Santoni FA, et al. Modelling and rescuing neurodevelopmental defect of Down syndrome using induced pluripotent stem cells from monozygotic twins discordant for trisomy 21. EMBO Mol Med. 2014;6: 259–277. doi:10.1002/emmm.201302848](https://www.zotero.org/google-docs/?iiTQ8H)

[2. Letourneau A, Santoni FA, Bonilla X, Sailani MR, Gonzalez D, Kind J, et al. Domains of genome-wide gene expression dysregulation in Down’s syndrome. Nature. 2014;508: 345–350. doi:10.1038/nature13200](https://www.zotero.org/google-docs/?iiTQ8H)

[3. Jin S, Lee YK, Lim YC, Zheng Z, Lin XM, Ng DPY, et al. Global DNA hypermethylation in down syndrome placenta. PLoS Genet. 2013;9: e1003515. doi:10.1371/journal.pgen.1003515](https://www.zotero.org/google-docs/?iiTQ8H)

[4. Do LH, Mobley WC, Singhal N. Questioned validity of Gene Expression Dysregulated Domains in Down’s Syndrome. F1000Research. 2015;4: 269. doi:10.12688/f1000research.6735.1](https://www.zotero.org/google-docs/?iiTQ8H)

[5. Gonzales PK, Roberts CM, Fonte V, Jacobsen C, Stein GH, Link CD. Transcriptome analysis of genetically matched human induced pluripotent stem cells disomic or trisomic for chromosome 21. PloS One. 2018;13: e0194581. doi:10.1371/journal.pone.0194581](https://www.zotero.org/google-docs/?iiTQ8H)

[6. Altug-Teber O, Bonin M, Walter M, Mau-Holzmann UA, Dufke A, Stappert H, et al. Specific transcriptional changes in human fetuses with autosomal trisomies. Cytogenet Genome Res. 2007;119: 171–184. doi:10.1159/000112058](https://www.zotero.org/google-docs/?iiTQ8H)

[7. Amano K, Sago H, Uchikawa C, Suzuki T, Kotliarova SE, Nukina N, et al. Dosage-dependent over-expression of genes in the trisomic region of Ts1Cje mouse model for Down syndrome. Hum Mol Genet. 2004;13: 1333–1340. doi:10.1093/hmg/ddh154](https://www.zotero.org/google-docs/?iiTQ8H)

[8. Dauphinot L, Lyle R, Rivals I, Dang MT, Moldrich RX, Golfier G, et al. The cerebellar transcriptome during postnatal development of the Ts1Cje mouse, a segmental trisomy model for Down syndrome. Hum Mol Genet. 2005;14: 373–384. doi:10.1093/hmg/ddi033](https://www.zotero.org/google-docs/?iiTQ8H)

[9. Lockstone HE, Harris LW, Swatton JE, Wayland MT, Holland AJ, Bahn S. Gene expression profiling in the adult Down syndrome brain. Genomics. 2007;90: 647–660. doi:10.1016/j.ygeno.2007.08.005](https://www.zotero.org/google-docs/?iiTQ8H)

[10. O’Doherty A, Ruf S, Mulligan C, Hildreth V, Errington ML, Cooke S, et al. An aneuploid mouse strain carrying human chromosome 21 with Down syndrome phenotypes. Science. 2005;309: 2033–2037. doi:10.1126/science.1114535](https://www.zotero.org/google-docs/?iiTQ8H)

[11. Mao R, Wang X, Spitznagel EL, Frelin LP, Ting JC, Ding H, et al. Primary and secondary transcriptional effects in the developing human Down syndrome brain and heart. Genome Biol. 2005;6: R107. doi:10.1186/gb-2005-6-13-r107](https://www.zotero.org/google-docs/?iiTQ8H)

[12. Slonim DK, Koide K, Johnson KL, Tantravahi U, Cowan JM, Jarrah Z, et al. Functional genomic analysis of amniotic fluid cell-free mRNA suggests that oxidative stress is significant in Down syndrome fetuses. Proc Natl Acad Sci U S A. 2009;106: 9425–9429. doi:10.1073/pnas.0903909106](https://www.zotero.org/google-docs/?iiTQ8H)

[13. De Cegli R, Romito A, Iacobacci S, Mao L, Lauria M, Fedele AO, et al. A mouse embryonic stem cell bank for inducible overexpression of human chromosome 21 genes. Genome Biol. 2010;11: R64. doi:10.1186/gb-2010-11-6-r64](https://www.zotero.org/google-docs/?iiTQ8H)

[14. Chou ST, Byrska-Bishop M, Tober JM, Yao Y, Vandorn D, Opalinska JB, et al. Trisomy 21-associated defects in human primitive hematopoiesis revealed through induced pluripotent stem cells. Proc Natl Acad Sci U S A. 2012;109: 17573–17578. doi:10.1073/pnas.1211175109](https://www.zotero.org/google-docs/?iiTQ8H)

[15. Cowley PM, Keslacy S, Middleton FA, DeRuisseau LR, Fernhall B, Kanaley JA, et al. Functional and biochemical characterization of soleus muscle in Down syndrome mice: insight into the muscle dysfunction seen in the human condition. Am J Physiol Regul Integr Comp Physiol. 2012;303: R1251-1260. doi:10.1152/ajpregu.00312.2012](https://www.zotero.org/google-docs/?iiTQ8H)

[16. MacLean GA, McEldoon J, Huang J, Allred J, Canver MC, Orkin SH. Downregulation of Endothelin Receptor B Contributes to Defective B Cell Lymphopoiesis in Trisomy 21 Pluripotent Stem Cells. Sci Rep. 2018;8: 8001. doi:10.1038/s41598-018-26123-y](https://www.zotero.org/google-docs/?iiTQ8H)

[17. Aziz NM, Guedj F, Pennings JLA, Olmos-Serrano JL, Siegel A, Haydar TF, et al. Lifespan analysis of brain development, gene expression and behavioral phenotypes in the Ts1Cje, Ts65Dn and Dp(16)1/Yey mouse models of Down syndrome. Dis Model Mech. 2018;11. doi:10.1242/dmm.031013](https://www.zotero.org/google-docs/?iiTQ8H)

[18. Moreira-Filho CA, Bando SY, Bertonha FB, Silva FN, Costa L da F, Ferreira LR, et al. Modular transcriptional repertoire and MicroRNA target analyses characterize genomic dysregulation in the thymus of Down syndrome infants. Oncotarget. 2016;7: 7497–7533. doi:10.18632/oncotarget.7120](https://www.zotero.org/google-docs/?iiTQ8H)

[19. Halevy T, Biancotti J-C, Yanuka O, Golan-Lev T, Benvenisty N. Molecular Characterization of Down Syndrome Embryonic Stem Cells Reveals a Role for RUNX1 in Neural Differentiation. Stem Cell Rep. 2016;7: 777–786. doi:10.1016/j.stemcr.2016.08.003](https://www.zotero.org/google-docs/?iiTQ8H)

[20. Bourquin J-P, Subramanian A, Langebrake C, Reinhardt D, Bernard O, Ballerini P, et al. Identification of distinct molecular phenotypes in acute megakaryoblastic leukemia by gene expression profiling. Proc Natl Acad Sci U S A. 2006;103: 3339–3344. doi:10.1073/pnas.0511150103](https://www.zotero.org/google-docs/?iiTQ8H)

[21. Olmos-Serrano JL, Kang HJ, Tyler WA, Silbereis JC, Cheng F, Zhu Y, et al. Down Syndrome Developmental Brain Transcriptome Reveals Defective Oligodendrocyte Differentiation and Myelination. Neuron. 2016;89: 1208–1222. doi:10.1016/j.neuron.2016.01.042](https://www.zotero.org/google-docs/?iiTQ8H)

[22. Bianco K, Gormley M, Farrell J, Zhou Y, Oliverio O, Tilden H, et al. Placental transcriptomes in the common aneuploidies reveal critical regions on the trisomic chromosomes and genome-wide effects. Prenat Diagn. 2016;36: 812–822. doi:10.1002/pd.4862](https://www.zotero.org/google-docs/?iiTQ8H)

[23. Bofill-De Ros X, Santos M, Vila-Casadesús M, Villanueva E, Andreu N, Dierssen M, et al. Genome-wide miR-155 and miR-802 target gene identification in the hippocampus of Ts65Dn Down syndrome mouse model by miRNA sponges. BMC Genomics. 2015;16: 907. doi:10.1186/s12864-015-2160-6](https://www.zotero.org/google-docs/?iiTQ8H)

[24. Byrska-Bishop M, VanDorn D, Campbell AE, Betensky M, Arca PR, Yao Y, et al. Pluripotent stem cells reveal erythroid-specific activities of the GATA1 N-terminus. J Clin Invest. 2015;125: 993–1005. doi:10.1172/JCI75714](https://www.zotero.org/google-docs/?iiTQ8H)

[25. Guedj F, Pennings JLA, Ferres MA, Graham LC, Wick HC, Miczek KA, et al. The fetal brain transcriptome and neonatal behavioral phenotype in the Ts1Cje mouse model of Down syndrome. Am J Med Genet A. 2015;167A: 1993–2008. doi:10.1002/ajmg.a.37156](https://www.zotero.org/google-docs/?iiTQ8H)

[26. Ling K-H, Hewitt CA, Tan K-L, Cheah P-S, Vidyadaran S, Lai M-I, et al. Functional transcriptome analysis of the postnatal brain of the Ts1Cje mouse model for Down syndrome reveals global disruption of interferon-related molecular networks. BMC Genomics. 2014;15: 624. doi:10.1186/1471-2164-15-624](https://www.zotero.org/google-docs/?iiTQ8H)

[27. Guedj F, Pennings JLA, Wick HC, Bianchi DW. Analysis of adult cerebral cortex and hippocampus transcriptomes reveals unique molecular changes in the Ts1Cje mouse model of down syndrome. Brain Pathol Zurich Switz. 2015;25: 11–23. doi:10.1111/bpa.12151](https://www.zotero.org/google-docs/?iiTQ8H)

[28. Weick JP, Held DL, Bonadurer GF, Doers ME, Liu Y, Maguire C, et al. Deficits in human trisomy 21 iPSCs and neurons. Proc Natl Acad Sci U S A. 2013;110: 9962–9967. doi:10.1073/pnas.1216575110](https://www.zotero.org/google-docs/?iiTQ8H)

[29. Volk M, Maver A, Lovrečić L, Juvan P, Peterlin B. Expression signature as a biomarker for prenatal diagnosis of trisomy 21. PloS One. 2013;8: e74184. doi:10.1371/journal.pone.0074184](https://www.zotero.org/google-docs/?iiTQ8H)

[30. Briggs JA, Sun J, Shepherd J, Ovchinnikov DA, Chung T-L, Nayler SP, et al. Integration-free induced pluripotent stem cells model genetic and neural developmental features of down syndrome etiology. Stem Cells Dayt Ohio. 2013;31: 467–478. doi:10.1002/stem.1297](https://www.zotero.org/google-docs/?iiTQ8H)

[31. Helguera P, Seiglie J, Rodriguez J, Hanna M, Helguera G, Busciglio J. Adaptive downregulation of mitochondrial function in down syndrome. Cell Metab. 2013;17: 132–140. doi:10.1016/j.cmet.2012.12.005](https://www.zotero.org/google-docs/?iiTQ8H)

[32. Li LB, Chang K-H, Wang P-R, Hirata RK, Papayannopoulou T, Russell DW. Trisomy correction in Down syndrome induced pluripotent stem cells. Cell Stem Cell. 2012;11: 615–619. doi:10.1016/j.stem.2012.08.004](https://www.zotero.org/google-docs/?iiTQ8H)

[33. Ripoll C, Rivals I, Ait Yahya-Graison E, Dauphinot L, Paly E, Mircher C, et al. Molecular signatures of cardiac defects in Down syndrome lymphoblastoid cell lines suggest altered ciliome and Hedgehog pathways. PloS One. 2012;7: e41616. doi:10.1371/journal.pone.0041616](https://www.zotero.org/google-docs/?iiTQ8H)

[34. Li C, Jin L, Bai Y, Chen Q, Fu L, Yang M, et al. Genome-wide expression analysis in Down syndrome: insight into immunodeficiency. PloS One. 2012;7: e49130. doi:10.1371/journal.pone.0049130](https://www.zotero.org/google-docs/?iiTQ8H)

[35. Lima FA, Moreira-Filho CA, Ramos PL, Brentani H, Lima L de A, Arrais M, et al. Decreased AIRE expression and global thymic hypofunction in Down syndrome. J Immunol Baltim Md 1950. 2011;187: 3422–3430. doi:10.4049/jimmunol.1003053](https://www.zotero.org/google-docs/?iiTQ8H)

[36. Loudin MG, Wang J, Leung H-CE, Gurusiddappa S, Meyer J, Condos G, et al. Genomic profiling in Down syndrome acute lymphoblastic leukemia identifies histone gene deletions associated with altered methylation profiles. Leukemia. 2011;25: 1555–1563. doi:10.1038/leu.2011.128](https://www.zotero.org/google-docs/?iiTQ8H)

[37. Pennings JLA, Rodenburg W, Imholz S, Koster MPH, van Oostrom CTM, Breit TM, et al. Gene expression profiling in a mouse model identifies fetal liver- and placenta-derived potential biomarkers for Down Syndrome screening. PloS One. 2011;6: e18866. doi:10.1371/journal.pone.0018866](https://www.zotero.org/google-docs/?iiTQ8H)

[38. Klusmann J-H, Godinho FJ, Heitmann K, Maroz A, Koch ML, Reinhardt D, et al. Developmental stage-specific interplay of GATA1 and IGF signaling in fetal megakaryopoiesis and leukemogenesis. Genes Dev. 2010;24: 1659–1672. doi:10.1101/gad.1903410](https://www.zotero.org/google-docs/?iiTQ8H)

[39. Hewitt CA, Ling K-H, Merson TD, Simpson KM, Ritchie ME, King SL, et al. Gene network disruptions and neurogenesis defects in the adult Ts1Cje mouse model of Down syndrome. PloS One. 2010;5: e11561. doi:10.1371/journal.pone.0011561](https://www.zotero.org/google-docs/?iiTQ8H)

[40. Hertzberg L, Vendramini E, Ganmore I, Cazzaniga G, Schmitz M, Chalker J, et al. Down syndrome acute lymphoblastic leukemia, a highly heterogeneous disease in which aberrant expression of CRLF2 is associated with mutated JAK2: a report from the International BFM Study Group. Blood. 2010;115: 1006–1017. doi:10.1182/blood-2009-08-235408](https://www.zotero.org/google-docs/?iiTQ8H)

[41. Klusmann J-H, Li Z, Böhmer K, Maroz A, Koch ML, Emmrich S, et al. miR-125b-2 is a potential oncomiR on human chromosome 21 in megakaryoblastic leukemia. Genes Dev. 2010;24: 478–490. doi:10.1101/gad.1856210](https://www.zotero.org/google-docs/?iiTQ8H)

[42. Moldrich RX, Dauphinot L, Laffaire J, Vitalis T, Hérault Y, Beart PM, et al. Proliferation deficits and gene expression dysregulation in Down’s syndrome (Ts1Cje) neural progenitor cells cultured from neurospheres. J Neurosci Res. 2009;87: 3143–3152. doi:10.1002/jnr.22131](https://www.zotero.org/google-docs/?iiTQ8H)

[43. Laffaire J, Rivals I, Dauphinot L, Pasteau F, Wehrle R, Larrat B, et al. Gene expression signature of cerebellar hypoplasia in a mouse model of Down syndrome during postnatal development. BMC Genomics. 2009;10: 138. doi:10.1186/1471-2164-10-138](https://www.zotero.org/google-docs/?iiTQ8H)

[44. Lepagnol-Bestel A-M, Zvara A, Maussion G, Quignon F, Ngimbous B, Ramoz N, et al. DYRK1A interacts with the REST/NRSF-SWI/SNF chromatin remodelling complex to deregulate gene clusters involved in the neuronal phenotypic traits of Down syndrome. Hum Mol Genet. 2009;18: 1405–1414. doi:10.1093/hmg/ddp047](https://www.zotero.org/google-docs/?iiTQ8H)

[45. Loe-Mie Y, Lepagnol-Bestel A-M, Maussion G, Doron-Faigenboim A, Imbeaud S, Delacroix H, et al. SMARCA2 and other genome-wide supported schizophrenia-associated genes: regulation by REST/NRSF, network organization and primate-specific evolution. Hum Mol Genet. 2010;19: 2841–2857. doi:10.1093/hmg/ddq184](https://www.zotero.org/google-docs/?iiTQ8H)

[46. Reinholdt LG, Czechanski A, Kamdar S, King BL, Sun F, Handel MA. Meiotic behavior of aneuploid chromatin in mouse models of Down syndrome. Chromosoma. 2009;118: 723–736. doi:10.1007/s00412-009-0230-8](https://www.zotero.org/google-docs/?iiTQ8H)

[47. Chou CY, Liu LY, Chen CY, Tsai CH, Hwa HL, Chang LY, et al. Gene expression variation increase in trisomy 21 tissues. Mamm Genome Off J Int Mamm Genome Soc. 2008;19: 398–405. doi:10.1007/s00335-008-9121-1](https://www.zotero.org/google-docs/?iiTQ8H)

[48. Conti A, Fabbrini F, D’Agostino P, Negri R, Greco D, Genesio R, et al. Altered expression of mitochondrial and extracellular matrix genes in the heart of human fetuses with chromosome 21 trisomy. BMC Genomics. 2007;8: 268. doi:10.1186/1471-2164-8-268](https://www.zotero.org/google-docs/?iiTQ8H)

[49. Wilson MD, Barbosa-Morais NL, Schmidt D, Conboy CM, Vanes L, Tybulewicz VLJ, et al. Species-specific transcription in mice carrying human chromosome 21. Science. 2008;322: 434–438. doi:10.1126/science.1160930](https://www.zotero.org/google-docs/?iiTQ8H)

[50. Costa V, Sommese L, Casamassimi A, Colicchio R, Angelini C, Marchesano V, et al. Impairment of circulating endothelial progenitors in Down syndrome. BMC Med Genomics. 2010;3: 40. doi:10.1186/1755-8794-3-40](https://www.zotero.org/google-docs/?iiTQ8H)

[51. Raveau M, Lignon JM, Nalesso V, Duchon A, Groner Y, Sharp AJ, et al. The App-Runx1 region is critical for birth defects and electrocardiographic dysfunctions observed in a Down syndrome mouse model. PLoS Genet. 2012;8: e1002724. doi:10.1371/journal.pgen.1002724](https://www.zotero.org/google-docs/?iiTQ8H)

[52. Granese B, Scala I, Spatuzza C, Valentino A, Coletta M, Vacca RA, et al. Validation of microarray data in human lymphoblasts shows a role of the ubiquitin-proteasome system and NF-kB in the pathogenesis of Down syndrome. BMC Med Genomics. 2013;6: 24. doi:10.1186/1755-8794-6-24](https://www.zotero.org/google-docs/?iiTQ8H)

[53. Ng AP, Hu Y, Metcalf D, Hyland CD, Ierino H, Phipson B, et al. Early lineage priming by trisomy of Erg leads to myeloproliferation in a Down syndrome model. PLoS Genet. 2015;11: e1005211. doi:10.1371/journal.pgen.1005211](https://www.zotero.org/google-docs/?iiTQ8H)

[54. Lane AA, Chapuy B, Lin CY, Tivey T, Li H, Townsend EC, et al. Triplication of a 21q22 region contributes to B cell transformation through HMGN1 overexpression and loss of histone H3 Lys27 trimethylation. Nat Genet. 2014;46: 618–623. doi:10.1038/ng.2949](https://www.zotero.org/google-docs/?iiTQ8H)

[55. Sullivan KD, Lewis HC, Hill AA, Pandey A, Jackson LP, Cabral JM, et al. Trisomy 21 consistently activates the interferon response. eLife. 2016;5. doi:10.7554/eLife.16220](https://www.zotero.org/google-docs/?iiTQ8H)

[56. Mowery CT, Reyes JM, Cabal-Hierro L, Higby KJ, Karlin KL, Wang JH, et al. Trisomy of a Down Syndrome Critical Region Globally Amplifies Transcription via HMGN1 Overexpression. Cell Rep. 2018;25: 1898-1911.e5. doi:10.1016/j.celrep.2018.10.061](https://www.zotero.org/google-docs/?iiTQ8H)

[57. Powers RK, Culp-Hill R, Ludwig MP, Smith KP, Waugh KA, Minter R, et al. Trisomy 21 activates the kynurenine pathway via increased dosage of interferon receptors. Nat Commun. 2019;10: 4766. doi:10.1038/s41467-019-12739-9](https://www.zotero.org/google-docs/?iiTQ8H)
