# Supplementary material for: Meta-analysis of transcriptomic data reveals clusters of consistently deregulated gene and disease ontologies in Down syndrome
Source: PLoS Comput Biol. 2021 Sep 27;17(9):e1009317. doi: 10.1371/journal.pcbi.1009317 (PMC8496798; doi:10.1371/journal.pcbi.1009317)
Supplement: S2 Table — (DOCX) [file pcbi.1009317.s012.docx]

**S2 Table.** Terms used in the meta-analysis

| **Comparison** | Differential expression analysis between two homogenous groups of samples, differing only in the disomic/trisomic status and blocking for other variables. |
| --- | --- |
| **Differentially expressed (DE) gene** | Gene found DE in at least one comparison |
| **HSA21 gene** | Gene mapping on HSA21 or mouse gene whose ortholog maps on HSA21 |
| **HSA21 interactors** | Genes reporting a high STRINGdb interaction score (>=900) with a HSA21 gene |
| **Number of occurrences** | Number of comparisons in which a gene is found DE |
| **Tissue macro-category** | One of the 9 categories in which we divided our heterogeneous datasets ("Blood/bone marrow", "Brain", "Endothelial", "Fibroblasts", "Heart", "Liver","Muscle", "Placenta/amnios", "Undifferentiated") |
| **Entropy** | Number of tissue macro-categories in which a gene is found DE. Higher score indicates more entropy |
| **Tissue specific gene** | Genes with an expression level greater than 1 (TPM or FPKM) that also have at least five-fold higher expression levels in a particular tissue (or group of tissues) compared to the average levels in all other tissues were defined as tissue specific, as defined in [[34]](https://www.zotero.org/google-docs/?glBTrR) |
| **Consistently DE gene** | Gene found in the 5% right tail of the distribution of occurrences of all the DE genes. This includes all genes found DE in at least 4 comparisons across all tissue macro-categories |
| **preferentially DE in a tissue macro-category** | Gene found in the 5% right tail of the distribution of occurrences of all the DE genes in only one given tissue macro-categories. This corresponds to genes DE in at least 4 comparisons for blood, 4 for brain, 3 for fibroblasts, and 2 for undifferentiated, placenta/amnios and liver [[36]](https://www.zotero.org/google-docs/?MrLkZx) |
| **DE gene across tissue macro-categories** | Gene found in the 5% right tail of the distribution of occurrences of all the DE genes in more than one tissue macro-categories. |
| **Top 500 genes** | The 500 genes that are consistently DE across tissue (all genes found DE in at least 4 comparisons across all tissue macro-categories) |
| **Co-DE gene pair** | Two genes DE in the same trisomic vs. disomic comparison |
| **Consistently co-DE gene pair** | Two genes of the top-500 DE in at least 4 common comparisons disregarding tissue macro category |
| **Consistently co-DE gene cluster** | Largest groups of genes of the top-500 genes which were consistently co-DE in at least 4 comparisons (we found two clusters of 13 and 11 genes) |
| **Pseudo-t-score** | Mean log2FC across comparisons divided by the standard deviation of the log2FC across comparisons over the square root of the number of comparisons. Only the comparisons where the gene is found DE are used in the computation of the mean value of that given gene. |
| **Consistently changing genes** | Genes consistently up- or down-regulated with a pseudo t-score on the 5% tails (>1.96 or <-1.96) |
| **Consistently up-regulated genes** | Consistently changing genes with positive pseudo t-score (>1.96) |
| **Consistently down-regulated genes** | Consistently changing genes with negative pseudo t-score (<-1.96) |
| **DS network** | Network where each node is one of the top-500 genes (HSA21, HSA21 neighbors and non-HSA21 genes) and edges represent consistently co-DE gene pairs |
